# Caprylic Acid (FFA C8:0) promotes the progression of prostate cancer by up-regulating G protein-coupled receptor 84/ Krüppel-like factor 7

**DOI:** 10.1186/s12885-023-10841-2

**Published:** 2023-05-11

**Authors:** Xue Li, Chenggang Yuan, Bingqi Yang, Huai Pang, Wei Li, Menghuan Li, Yihan Tang, Dingling Ma, Jianxin Xie, Jingzhou Wang, Jun Zhang

**Affiliations:** 1 Shihezi University School of Medicine, Bei-Er-Lu, Shihezi, 832000 Xinjiang China; 2 Institute of Clinical Medicine, Zhanjiang Central People’s Hospital, Zhanjiang, 524045 China; 3 Laboratory of Xinjiang Endemic and Ethic Diseases, Shihezi University, Shihezi, 832000 Xinjiang China

**Keywords:** Caprylic Acid, GPR84, KLF7, IL-6, p21, Prostate cancer

## Abstract

**Background:**

In previous study, we found that the content of medium-chain fatty acid Caprylic Acid (FFA C8:0) may be an important risk factor of obesity induced prostate cancer (PCa). However, the relationship between FFA C8:0 and PCa has not been reported. In this study, we explored whether the FFA C8:0 can promotes the progression of PCa by up-regulating Krüppel-like factor 7 (KLF7).

**Methods:**

We collected tissues from PCa patients and Benign Prostate Hyperplasia (BPH), constructed a primary-tumor bearing mouse model with obesity through high-fat diet, and observed the tumor formation ability of PCa cells. In vitro, CCK8 assay, plate cloning, Transwell and scratch experiment were used to detect the changes in biological behavior of PCa cells stimulated by FFA C8:0.

**Results:**

First, we found that the expression level of KLF7 is higher in PCa tissues of patients, and the expression of KLF7 is positively correlated with tumour-promoting gene IL-6, while it is negative correlated with another tumour-suppressor gene p21. Then, this study found that PCa cells were more likely to form tumors in diet induced obese mice. Compared with the normal diet group (ND), the expression levels of KLF7 in tumor tissues in high-fat diet group (HFD) were higher. Futhermore, we verified that high concentrations of FFA C8:0 can promote the biological behavior of PCa cells by activating KLF7/IL-6/p21 signaling pathway, which is mediated by the GPR84.

**Conclusions:**

Our research may provide a potential target for clinical prevention and treatment of PCa which induced by obesity.

**Supplementary Information:**

The online version contains supplementary material available at 10.1186/s12885-023-10841-2.

## Background

As a male malignant tumor that ranks second in new incidence and fifth in mortality in the world, the occurrence and development of prostate cancer (PCa) are affected by many factors [1, 2]. Excluding the influence of family inheritance and age, obesity is significantly related to the risk of PCa [3]. A large number of epidemiological studies have pointed out that obesity is significantly related to the occurrence of aggressive and fatal PCa, the same is the recurrence of PCa after surgery [4–6]. Despite the development of anti-androgens therapy, radiotherapy, and surgery, the unclear molecular mechanism remains a major challenge in treating advanced PCa under obesity.

The results of recent research have shown that free fatty acids (FFAs) metabolism disorders play a key role in the pathogenesis of obesity [7]. Numerous studies have revealed that serum FFAs levels are elevated in PCa patients [8–11]. A prospective nutrition study showed that the intake of butyric acid is positively associated with advanced PCa [12]. In addition, arachidonic acid can promote the castration resistance of prostate cancer by inducing androgen production in steroid-starved prostate cancer cells, and oleic acid promotes an aggressive phenotype in PCa cells via calcium, and PI3K/Akt signaling [13, 14]. FFAs can be divided into short chain fatty acids, medium chain fatty acids and long chain fatty acids according to the length of carbon chain. Different kinds of FFAs act by activating different fatty acid receptors, among them, short-chain FFAs activate GPR41/43, medium-chain FFAs activate GPR84, and long-chain FFAs activate GPR40/120 [15, 16]. In previous studies, we found that the content of medium-chain FFAs caprylic acid (FFA C8:0) in the serum of patients with PCa and bone metastasis of PCa was significantly higher than that of non-cancerous individuals, it suggests that FFA C8:0 may play an important role in the progression of PCa, and elucidating its specific molecular mechanism will provide a novel target for the prevention and clinical treatment of PCa [17].

Kruppel-like factors (KLFs) are important transcription factors that regulate tumor development and lipid metabolism, and are involved in the regulation of cell differentiation, proliferation, angiogenesis and other signal patyways [18]. Among them, KLF7 is up-regulated in various cancer tissues, can act as an oncogene to promote the proliferation and metastasis of cancer cells [19–22]. However, whether KLF7 is inovlved in the process that FFA C8:0 promotes the progression of PCa remains to be explored. Moreover, high concentration of palmitic acid can activate IL-6 through KLF7 transcription and induce the inflammatory response of adipocytes [23]. In addition, as a key effector downstream of the tumor suppressor P53, p21 plays an inhibitory role in a variety of tumors.And it has been proven that KLF7 can transcriptionally control the expression of p21 by binding to its promoter region [24–27]*.*

In this study, we determined the expression levels of GPR84, KLF7, IL-6, p21 in tumor tissues of patients with PCa and obesity with primary PCa tumor-bearing mouse model. We also detected the biological behavior changes and the expression levels of GPR84, KLF7, IL-6, p21 under FFA C8:0 treatment of PCa cells (PC-3/22RV1) in *vitro*. Our study will provide a molecular target for the prevention and treatment of PCa in the clinic.

## Methods

### Human samples and measurement of biochemical indices

From March 2018 to May 2020, we collected tissues, serum and general data of of Benign Prostate Hyperplasia (BPH, *n* = 30) and PCa patients (*n* = 30) at Shihezi People's Hospital. The prostate tissue from BPH individuals and the tumor tissue from PCa patients were obtained by surgical puncture, some were embedded in paraffin wax for immunohistochemical detection, and some were frozen at -80 °C for extraction of protein and mRNA. All blood was collected on an empty stomach in the morning, centrifuged at 4000 RPM for 5 min, and the serum was drawn and frozen at -80 °C for detection of blood lipid. General information includes age, height, weight and body mass index (BMI = body weight (kg)/height (m)^2^). All experiments were performed in accordance with the protocol approved by the Medical Ethics Committee at First Affiliated Hospital, Shihezi University School of Medicine (reference number: 2017–049-01). All clinical patients participating in the study signed the informed consent.

## Animals

Fifteen 4-week-old male mice were raised in the specific pathogen free animal room (BALB/c-nu, from Vital River, Beijing, China). All male mice were randomly assigned to Test group and Control group before group feeding, and then fed in groups of 5 in a cage. Among them, 10 mouse in Test group were randomly assigned to 2 cages and fed with high fat. All food and drinking water are strictly sterilized and free to ingest. After a week of adaptive feeding, the mice were fed High-Fat Diet (HFD, Medicine, Jiangsu, China, Test group, *n* = 10) and Normal Diet (ND, Medicine, Jiangsu, China, Control group, *n* = 5). After the two groups of mice showed significant differences in body weight and Lee`s, 5 × 10^5^ PC3-Luc cells were injected into the prostate of each mice [28]. Mice were included in the study if they survived surgery after in-situ injection of PC3 cells into the prostate. Mice that died or were unable to eat or move normally after surgery were excluded prematurely.

HFD feeding continued for 12 weeks after surgery, they were given inhalation anesthetized using isoflurane, inject D-luc substrate enzyme into the abdominal cavity and observe the PCa tumor formation within 30 min using a small animal in vivo imaging instrument, cervical dislocation method made mice die and surgically felt the intra-abdominal tumor tissue. Four researchers participated in the animal study. The first researcher was responsible for group feeding and numbering of the mice, the second researcher was responsible for in-situ prostate injection surgery (the group of mice was unknown), the third researcher was responsible for vivo imaging and surgical dissection of tumor tissue from the mice (the group of mice was unknown), the fourth researcher was responsible for analyzing the tumor volume, weight and expression levels of various factors in the tissue according to the number (the group of mice was unknown), and sent the data to the first researcher for analysis.

All experiments involving mice were performed in accordance with the protocol approved by the Medical Ethics Committee at First Affiliated Hospital, Shihezi University School of Medicine (reference number: A2017-115–01). We determine that our manuscript reporting adheres to the ARRIVE guidelines. Detailed procedures have been placed in the Supplementary file 3 ARRIVE checklist.

### Immunohistochemistry

After dewaxing the section of paraffin tissue, repair the antigen in citric acid buffer under high temperature and pressure. After returning to room temperature, place the section in 3% hydrogen peroxide to remove excess peroxidase, and wash with 1 × PBS buffer. Each section was dripped with 1:200 KLF7 antibody (abcam, ab197690), 1:400 IL-6 antibody (abcam, ab6672), 1:200 p21 antibody (abcam, ab109520), and 1:400 Ki67 antibody (CST, 9449). After incubating for 12 h at 4 °C, return the sections to room temperature, wash with 1 × PBS buffer, add anti-mouse/rabbit HRP secondary antibody (DAKO) and incubate at 37℃ for 30 min, wash with 1 × PBS buffer, drop DAB chromogenic solution to observe the staining results. The scores were evaluated by two pathologists in the First Affiliated Hospital of Shihezi University School of Medicine. The final total score is equal to the percentage of positive cells(0–9%, 0; 10–19%, 1; 20–29%, 2; 30–39%, 3; 40–49%, 4; 50–59%, 5; 60–69%, 6; 70–79%, 7; 80–89%, 8; 90–100%, 9) multiplied by the positive staining intensity(negative, 0; yellow, 1; brownish yellow, 2; brown, 3).

### Biochemical indicator test

Levels of TG, TC, HDL-C, LDL-C, and glucose were measured by TG assay kit (Nanjing Jiancheng Bioengineering Institute, A110-1–1), TC assay kit (Nanjing Jiancheng Bioengineering Institute, A111-1–1), HDL-C assay kit (Nanjing Jiancheng Bioengineering Institute, A112-1–1), LDL-C assay kit (Nanjing Jiancheng Bioengineering Institute, A113-1–1), and glucose assay Kit (Nanjing Jiancheng Bioengineering Institute, F006-1–1).

### Reagents and materials

40 mM FFA C8:0 solution: FFA C8:0 (Solarbio, P5585, 38.04μL) was added to 3 mL NaOH solution (0.1 mol/L), placed in a 75 °C full saponification water bath for 30 min until the FFA C8:0 are completely dissolved and the liquid is colorless and transparent. Then the liquid was added to 3 mL BSA (Solarbio, A8850; 40%, free of fatty acid) solution immediately with sufficient mixing. 10 mM GPR84 antagonist 8 solution (MedChemExpress, HY-112562, 5 mg): was dissolved in 1.1864 mL DMSO.

### Cell lines and culture conditions

PC3 cells were purchased from Shanghai Cell Bank of the Chinese Academy of Sciences, cell catalog number: SCSP-532. PC3 cells were cultured in F12 medium (Gibco, 11,765,054) containing 10% fetal bovine serum + 1% penicillin–streptomycin mixture in an incubator at 37 °C, 5% CO_2_, and saturated humidity. 22RV1 cells were purchased from Shanghai Cell Bank of the Chinese Academy of Sciences, cell catalog number: SCSP-5022. 22RV1 cells were cultured in RPMI 1640 medium (Gibco, 61,870,036) containing 10% fetal bovine serum + 1% penicillin–streptomycin mixture in an incubator at 37 °C, 5% CO_2_, and saturated humidity.

### Western blot

Use 1% PMSF-containing RIPA lysis buffer to extract the total protein of the cells. After matching the protein concentration, add 4 × SDS-PAGE loading buffer and stop the enzymatic reaction in a dry bath at 100 °C for 10 min. After separating the protein in an acrylamide gel, the protein was transferred to a nitrocellulose membrane, and the membrane was blocked with bovine serum albumin for 3 h. Drop 1:1000 KLF7 antibody (abcam, ab197690), 1:1000 IL6 antibody (abcam, ab6672), 1:1000 p21 antibody (abcam, ab109520), 1:1000 MMP2 antibody (abcam, ab92536), and 1:1000 β-Tubulin antibody (Zhongshan Jinqiao, TA-10) and incubate for 12 h at 4℃. After TBST washes away excess antibody, drop 1: 10,000 s antibody and incubate for 2 h at room temperature. After TBST washes away excess antibody, the chemiluminescence reagent is droped and detected in ChemiScope mini-imaging system.

### Quantitative real‐time PCR

Use the TRIzol lysis method to extract total cellular mRNA in a 4 °C environment, and reverse-transcribe the mRNA into cDNA at 42 °C for 1 h, 70 °C for 15 min, and 4 °C for 30 min. Use QuantiNovaTM SYBR Green PCR Kit to detect mRNA expression level in 95 °C for 3 min, 45 cycles at 95 °C for 10 s, and 60 °C for 30 s. GAPDH was used as an internal control. Data were obtained as Ct values, and the 2^−ΔCt^ (ΔCt = single target gene Ct – sample GAPDH Ct) method was used in the analysis. Gene expression was quantified using a relative method. Supplementary Table 1 shows the primer sequences used.

### Cell Counting Kit‐8 (CCK8) assay

Digest and centrifuge the PCa cells confluent to 80% ~ 90% and inoculate the resuspension solution (PC3: 5 × 10^3^/well, 22RV1: 1 × 10^4^/well) in 96-well plate with a total volume of 100μL, After 24 h, perform group stimulation, add CCK8 (DOJINDO LABORATORIES, CK04) at 0, 24, 48, 72, 96, and 120 h and incubate at 37 °C for 3 h in the dark. Use a microplate reader (Bio-rad) to detect the OD value at 450 nm wavelength.

### Cell colony formation experiment

Digest and centrifuge the PCa cells confluent to 80% ~ 90% and inoculate the resuspension solution (PC3: 600/well, 22RV1: 600/well) in 6-well plate with a total volume of 2 mL and incubated at 37 °C, 5% CO_2_, and saturated humidity. After 24 h, group processing is performed, and the medium is changed every 2 days. Fix the cells with 4% neutral formaldehyde for 30 min on the 10th day, wash away impurities in the chamber, and add 1 mL 0.01% crystal violet staining solution to stain for 20 min.

### Cell invasion assay

Before the experiment, Matrigel Basement Membrane Matrix (Solarbio, 356,234; Matrigel: serum-free medium = 1:8) was evenly spread on the bottom of the Transwell chamber (pore size = 8.0 μm) at 4 °C, and incubated at 37 °C for 30 min. The cells of each group were digested and centrifuged, resuspended in serum-free medium, and spread in the upper chamber of Transwell (PC3: 1 × 10^5^/well, 22RV1: 1 × 10^5^/well). Add a medium containing fetal bovine serum (10%) to the lower chamber and incubate for 24 h in an incubator at 37 °C, 5% CO_2_, and saturated humidity. After fixing the cells with 4% neutral formaldehyde for 30 min, wash away impurities in the chamber, and add 500 μL 0.01% crystal violet staining solution to stain for 20 min. The mean average value was calculated based on nine different observation fields under 200 magnification.

### Cell migration assay

The cells of each group were digested and centrifuged, resuspended in serum-free medium, and spread in the upper chamber of Transwell (PC3: 1 × 10^5^/well, 22RV1: 1 × 10^5^/well). Add a medium containing fetal bovine serum (10%) to the lower chamber and incubate for 24 h in an incubator at 37 °C, 5% CO_2_, and saturated humidity. After fixing the cells with 4% neutral formaldehyde for 30 min, wash away impurities in the chamber, and add 500 μL 0.01% crystal violet staining solution to stain for 20 min. The mean average value was calculated based on nine different observation fields under 200 magnification.

### Cell scratch test

Digest and centrifuge PCa cells and evenly plant them in a 6-well plate. When the cells are more than 95% confluent, cells were incubated with complete medium containing Mitomycin (1 μg/mL) for 1 h, use a sterile pipette tip to scribble evenly in the cells along the diameter of the hole, wash the cells with 1 × PBS and take pictures under the microscope. At this time, the grouping process was initiated. After 24 or 48 h, the same position was photographed under the microscope to analyze the healing ability of the cells.

### Statistical analysis

SPSS (v. 17.0) computer software was used for all statistical analysis. Mean and standard deviation were determined as the main parameters, and the average of data between the experimental and control groups were compared using independent samples *t*-test, *one-way ANOVA* test and *nonparametric rank sum* test. Values of *P* < 0.05 as a standard of significant difference.

## Results

### GPR84/KLF7 is highly expressed in prostate cancer

**Fig. 1:**
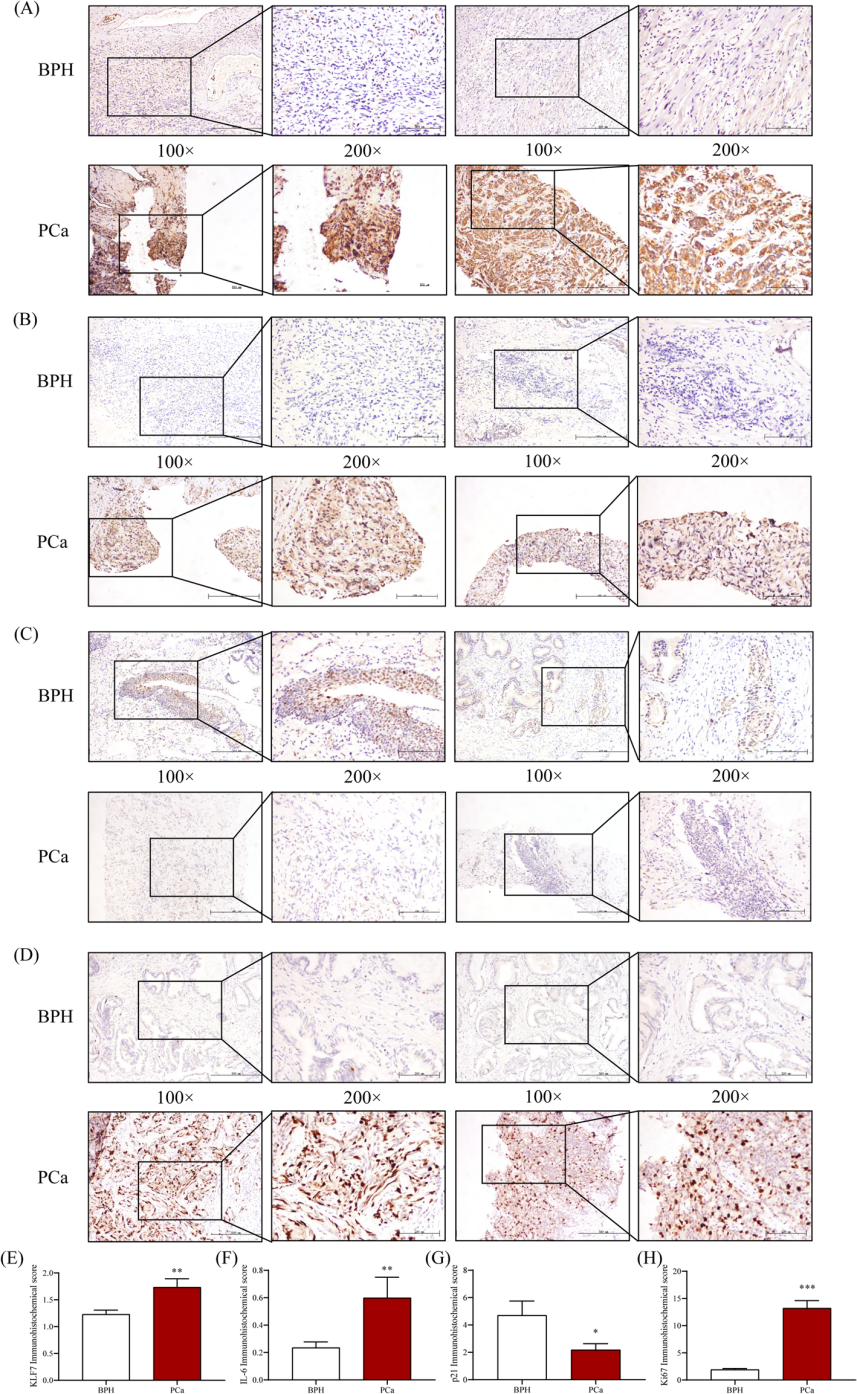
The expression levels of KLF7/ IL-6/ p21/ Ki67 in tumor tissues of patients with PCa**.** **A**-**D** Immunohistochemistry was used to detect the expression level of KLF7/ IL-6/ p21/ Ki67 in BPH tissues(up) and tumor tissues with PCa(down) (100 × , 200 ×). **E**–**H** Immunohistochemical score of KLF7/ IL-6/ p21/ Ki67. *t* test, *P < 0.05, **P < 0.01, ***P < 0.001 the difference was statistically significant

As shown in Supplementary Table 2, the serum prostate-specific antigen (PSA) level of patients with PCa is significantly higher than that of individuals with benign prostatic hyperplasia (BPH). In addition, the Triglyceride (TG) level of patients with PCa is significantly higher than that of BPH. Immunohistochemical showed that the expression level of KLF7 protein in the tumor tissues of patients with PCa was significantly higher than that of BPH (Fig. 1A, E). The protein and mRNA expression levels of KLF7 in the tumor tissues of PCa patients were also higher than those of BPH (Supplementary Fig. 1A-B). Besides, the mRNA expression level of GPR84 in tumor tissues of PCa patients was also significantly increased (Supplementary Fig. 1C).

Then, immunohistochemistry was used to detect the expression level of IL-6/ p21, possible downstream factors of KLF7. The result showed that the expression level of IL-6 in PCa tissue was significantly higher than that of BPH (Fig. 1B, F). In contrast, the expression level of the tumor-suppressor p21 was significantly lower in PCa tissue than that of BPH (Fig. 1C, G). Moreover, the expression level of Ki67 in PCa tissues was also significantly increased (Fig. 1D, H). The expression of KLF7 was positively correlated with the expression levels of PSA, IL-6, Ki67, and negatively correlated with the expression level of p21 (Supplementary Fig. 1D-J). The above results suggest that KLF7 may play an important function in the development of PCa.

**Fig. 2:**
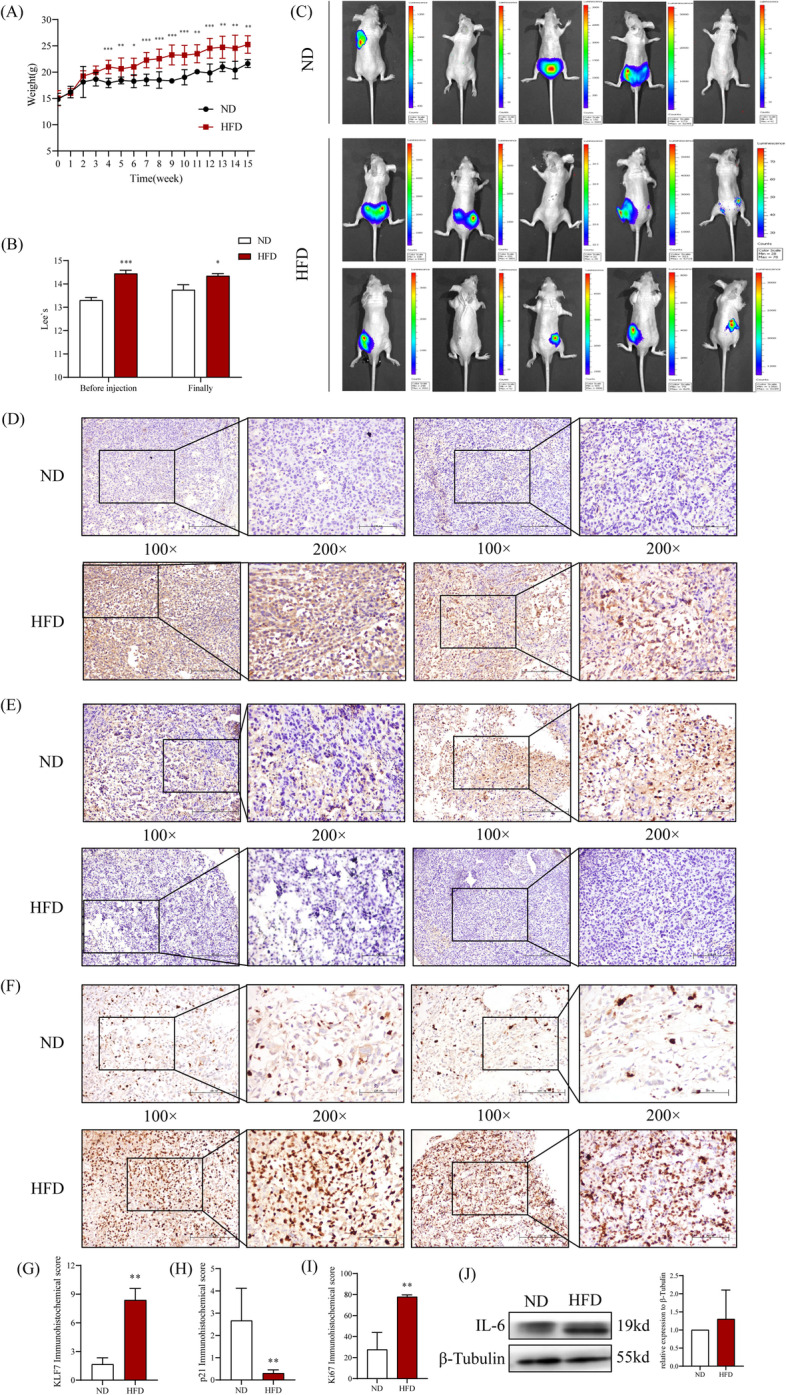
Tumor-forming ability of PCa cells in prostate and the expression level of KLF7 in tumor tissues under obesity. **A**-**B** The BALB/c nude mice were fed with 60% high fat diets, the weight (**A**) and Lee’s index (**B**) were detected. **C** The tumor-forming ability of PC3 cells in the prostates of the two groups of mice was observed by small animal imaging. **D**-**F** Immunohistochemistry was used to detect the expression levels of KLF7 (**D**), p21 (**E**) and Ki67 (**F**) in tumer tissues from ND group(up) and HFD group(down) (100 × , 200 ×). **G**-**I** Immunohistochemical score of KLF7 (**G**), p21 (**H**) and Ki67 (**I**). **J** Western Blot was used to detect the protein expression level of IL-6 in tumor tissues from ND group and HFD group. Non-parametric rank sum test, *P < 0.05, **P < 0.01, ***P < 0.001 the difference was statistically significant

To investigate whether the high expression of KLF7 in PCa tumors is related to obesity-induced PCa, male BALB/ C nude mouse were fed with high-fat diet (HFD, 60% fat Kcal%) to construct a mouse obesity model. After feeding for 4 weeks, the weight and Lee's index of HFD mice were significantly higher than those of the normal control diet group (ND, 10% fat Kcal%, Fig. 2A, B). PC3 cells were then injected into the prostate of each mouse. Finally, we found that PC3 cells are more likely to form tumors in mice fed with HFD (Supplementary Table 3, Fig. 2C). The contents of FFA, TG and TC in the serum of mice fed with HFD were significantly higher than those in ND mice (Supplementary Table 4). Furthermore, the expression levels of KLF7, IL-6, and Ki67 in PCa tissues of HFD mice were higher than ND mice, while p21 was the opposite (Fig. 2D-J). The above results suggest that KLF7 may play a considerable role in obesity-induced PCa, but its specific mechanism is still unclear.

### High concentration of FFA C8:0 can enhance the biological behavior of PCa cells

**Fig. 3:**
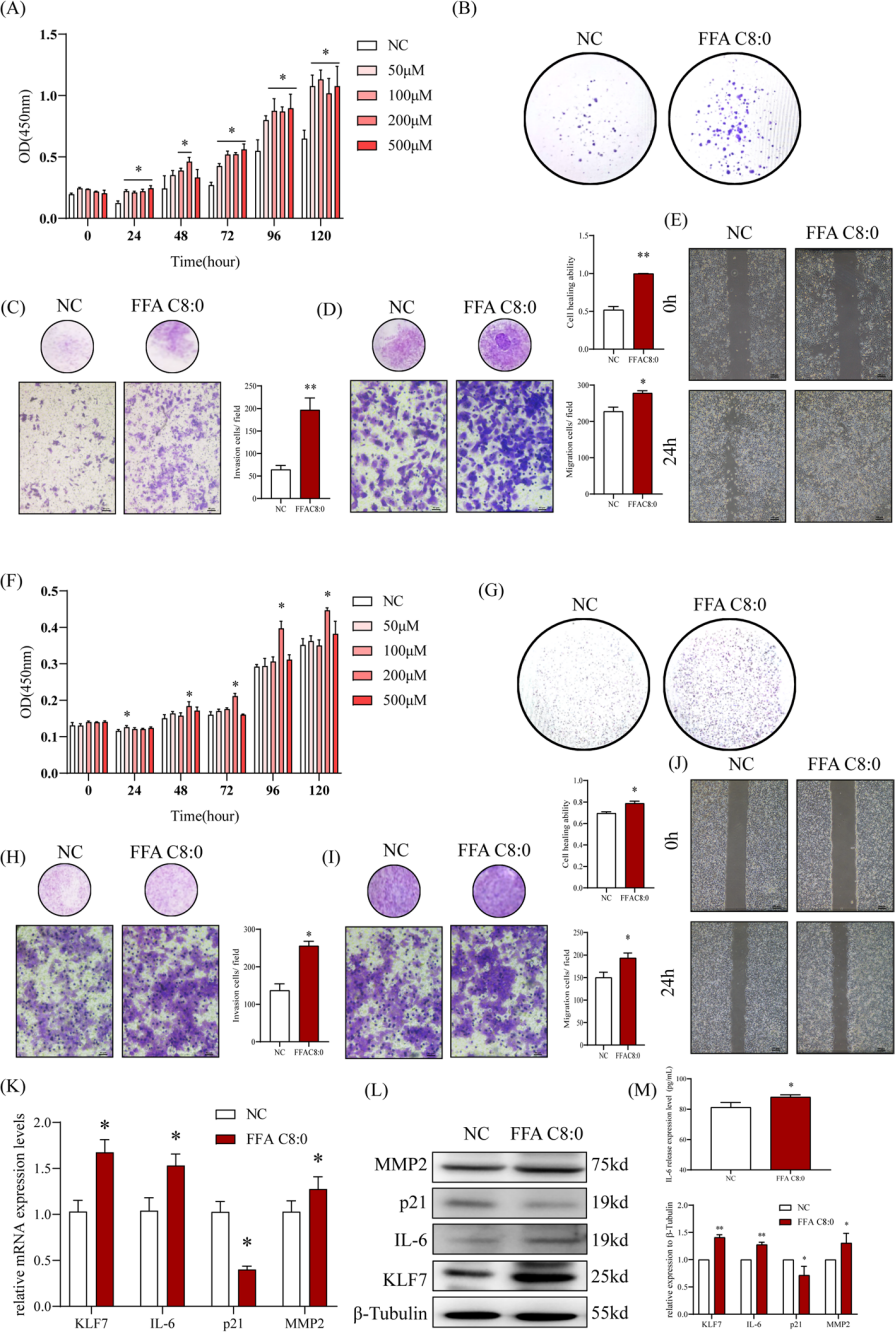
Effects of 200 μM FFA C8:0 on biological behavior and KLF7 expression level of PCa cells. **A**, **F** CCK8 assay was used to detect the proliferation abilities of PC3 cells (**A**) and 22RV1 cells (**F**) with different concentrations of FFA C8:0. **B**, **G** The effects of 200 μM FFA C8:0 on the colony formation ability of PC3 cells (**B**) and 22RV1 cells (**G**) were detected by plate Colony assay. **C**, **H** The effects of 200 μM FFA C8:0 on the invasion abilities of PC3 cells (**C**) and 22RV1 cells (**H**) were detected by Transwell assay (200 ×). **D**, **I** The effects of 200 μM FFA C8:0 on the migration abilities of PC3 cells (**D**) and 22RV1 cells (**I**) were detected by Transwell assay (200 ×). **E**, **J** The effects of 200 μM FFA C8:0 on the healing abilities of PC3 cells (**E**) and 22RV1 cells (**J**) were detected by scratch experiment (40 ×). **K **The mRNA expression levels of KLF7, IL-6, p21 and MMP2 in PC3 cells stimulated by 200 μM FFA C8:0 were detected by qRT-PCR. **L** The protein expression levels of KLF7, IL-6, p21 and MMP2 in PC3 cells stimulated by 200 μM FFA C8:0 were detected by Western Blot. **M** The secretion level of IL-6 in PC3 cells stimulated by 200 μM FFA C8:0 were detected by ELISA. *t* test, *P < 0.05, **P < 0.01 the difference was statistically significant

Two PCa cells PC3 (highly malignant, non-androgen-dependent) and 22RV1 (low-malignant, androgen-dependent) were treated with different concentrations of FFA C8:0, the results showed that high concentrations of FFA C8:0 can significantly promote the proliferation ability of two types of cells, and PC3 cells are more sensitive to FFA C8:0 (Fig. 3A, F). The colony-formation experiment indicated that high concentrations of FFA C8:0 can significantly enhance the colony forming ability of PC3 cells and 22RV1 cells (Fig. 3B, G). Transwell assays also showed that FFA C8:0 can significantly increase the invasion and migration ability of the two types of cells (Fig. 3C, D, H, I). Similarly, the scratch experiment confirmed that the healing ability of the two cell lines was significantly enhanced under high concentration of FFA C8:0 (Fig. 3E, J). The expression levels of KLF7, IL-6, and MMP2 increased in PC3 cells after being treated by FFA C8:0, while the expression level of p21 decreased (Fig. 3K-M).

### High concentration of FFA C8:0 can enhance the biological behavior of PCa cells by up-regulating KLF7

**Fig. 4:**
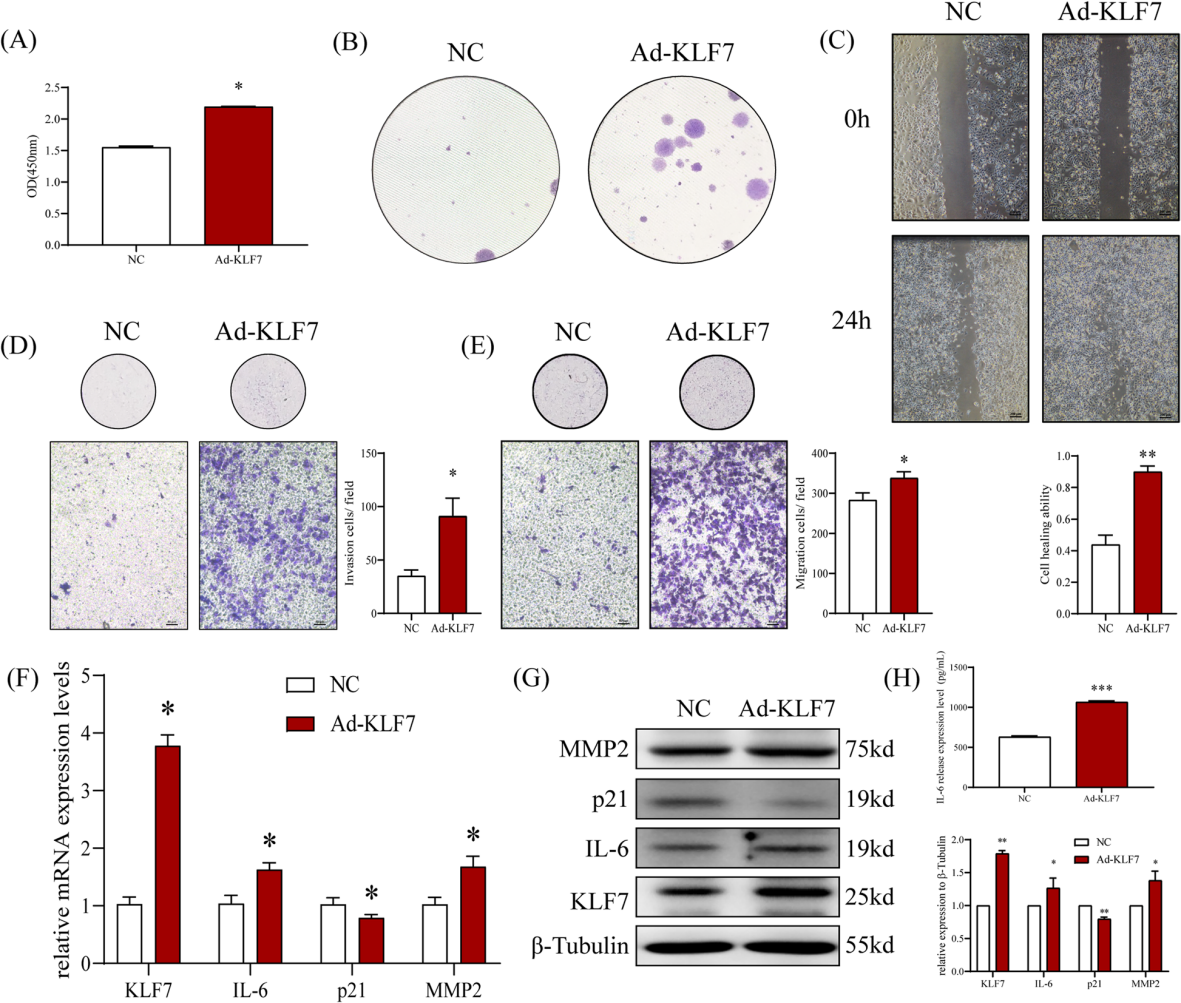
Effects of up-regulated KLF7 on biological behavior of PC3 cells. **A** CCK8 assay was used to detect the proliferation ability of PC3 cells after up-regulation of KLF7. **B** The effect of up-regulated KLF7 on the colony formation ability of PC3 was detected by plate Colony assay. **C** The effect of up-regulated KLF7 on the healing ability of PC3 cells was detected by scratch experiment (40 ×). **D**-**E** The effect of up-regulated KLF7 on the invasion (**D**) and migration (**E**) ability of PC3 cells was detected by Transwell assay (200 ×). **F** The mRNA expression levels of KLF7, IL-6, p21 and MMP2 in PC3 cells after up-regulating KLF7 were detected by qRT-PCR. **G** The protein expression levels of KLF7, IL-6, p21 and MMP2 in PC3 cells after up-regulating KLF7 were detected by Western Blot. **H** The secretion level of IL-6 in PC3 cells after up-regulating KLF7 were detected by ELISA. *t* test, *P < 0.05, **P < 0.01 the difference was statistically significant

**Fig. 5:**
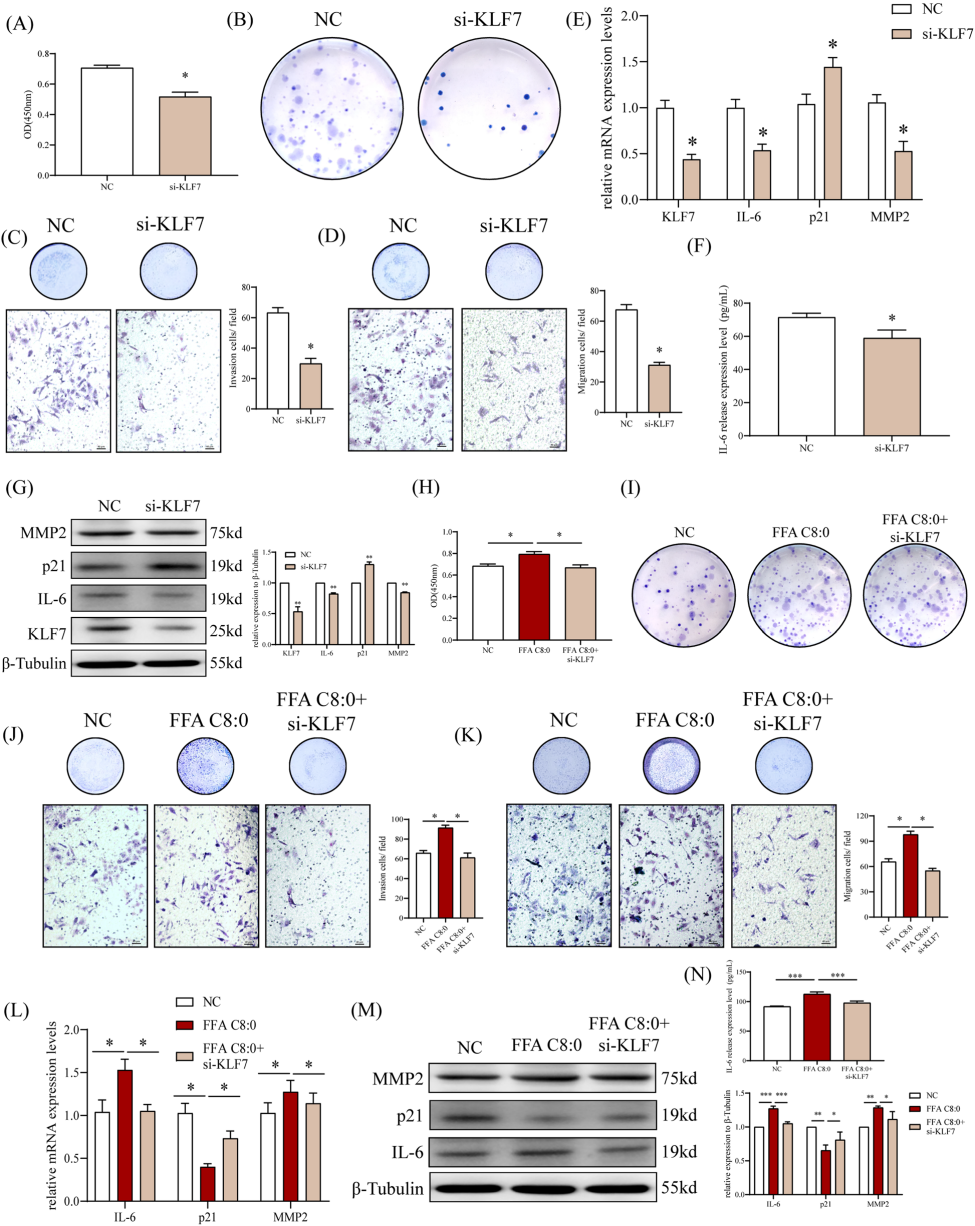
Effects of down-regulated KLF7 under 200 μM FFA C8:0 stimulation on biological behavior of PC3 cells. **A** CCK8 assay was used to detect the proliferation ability of PC3 cells after down-regulation of KLF7. **B** The effect of down-regulated KLF7 on the colony formation ability of PC3 was detected by plate Colony assay. **C**-**D** The effect of down-regulated KLF7 on the invasion **(C)** and migration (**D**) ability of PC3 cells was detected by Transwell assay (200 ×). **E** The mRNA expression levels of KLF7, IL-6, p21 and MMP2 in PC3 cells after down-regulating KLF7 was detected by qRT-PCR. **F** The secretion level of IL-6 in PC3 cells after down-regulating KLF7 were detected by ELISA. **G** The protein expression levels of KLF7, IL-6, p21 and MMP2 in PC3 cells after down-regulating KLF7 were detected by Western Blot. **H** CCK8 assay was used to detect the proliferation ability of PC3 cells after down-regulated KLF7 under 200 μM FFA C8:0 stimulation. **I** The effect of down-regulated KLF7 under 200 μM FFA C8:0 stimulation on the colony formation ability of PC3 was detected by plate Colony assay. **J**-**K** The effect of down-regulated KLF7 under 200 μM FFA C8:0 stimulation on the invasion (**J**) and migration (**K**) ability of PC3 cells was detected by Transwell assay (200 ×). **L** The mRNA expression levels of IL-6, p21 and MMP2 in PC3 cells after down-regulated KLF7 under 200 μM FFA C8:0 stimulation were detected by qRT-PCR. **M** The protein expression levels of IL-6, p21 and MMP2 in PC3 cells after down-regulated KLF7 under 200 μM FFA C8:0 stimulation were detected by Western Blot. **N** The secretion level of IL-6 in PC3 cells after down-regulated KLF7 under 200 μM FFA C8:0 stimulation were detected by ELISA. *t* test, *P < 0.05 the difference was statistically significant

It was confirmed that high concentration of FFA C8:0 can promote the biological behavior of PCa cells, and the expression levels of KLF7 and downstream factors are significantly increased after stimulated by FFA C8:0, then we want to verify whether FFA C8:0 promotes the development of PCa by up-regulating the expression of KLF7. First, KLF7 over-expression plasmid was transfected into PC3 cells, results showed that the proliferation, colony formation, invasion, migration, and scratch healing capabilities of PC3 cells were considerably enhanced (Fig. 4A-E). The expression levels of IL-6 and MMP2 in PC3 cells were increased and the expression level of p21 was inhibited after over-expression of KLF7 (Fig. 4F-H). After down-regulating KLF7 by using RNA interference assay, the proliferation, colony formation, invasion, and migration capabilities of PC3 cells were significantly weakened(Fig. 5A-D), at the same time, the expression levels of IL-6 and MMP2 were significantly reduced, while p21 was the opposite (Fig. 5E-G). The above results confirms that KLF7 may promote the development of PCa by regulating the expression of IL-6 and p21.

FFA C8:0 stimulated PC3 cells while down-regulated the expression of KLF7, we found that both the proliferation, colony formation, invasion, and migration capabilities of PC3 cells were suppressed (Fig. 5H-K). Similarly, the expression levels of IL-6 and MMP2 were significantly inhibited, while p21 was the opposite (Fig. 5L-N). Therefore, we confirm that FFA C8:0 promotes the biological behavior of PCa cells by up-regulating the expression of KLF7. However, the mechanism that FFA C8:0 affects the expression of KLF7 is still to be explored.

### High concentration of FFA C8:0 can up-regulate KLF7 and enhance the biological behavior of PCa cells via GPR84

**Fig. 6:**
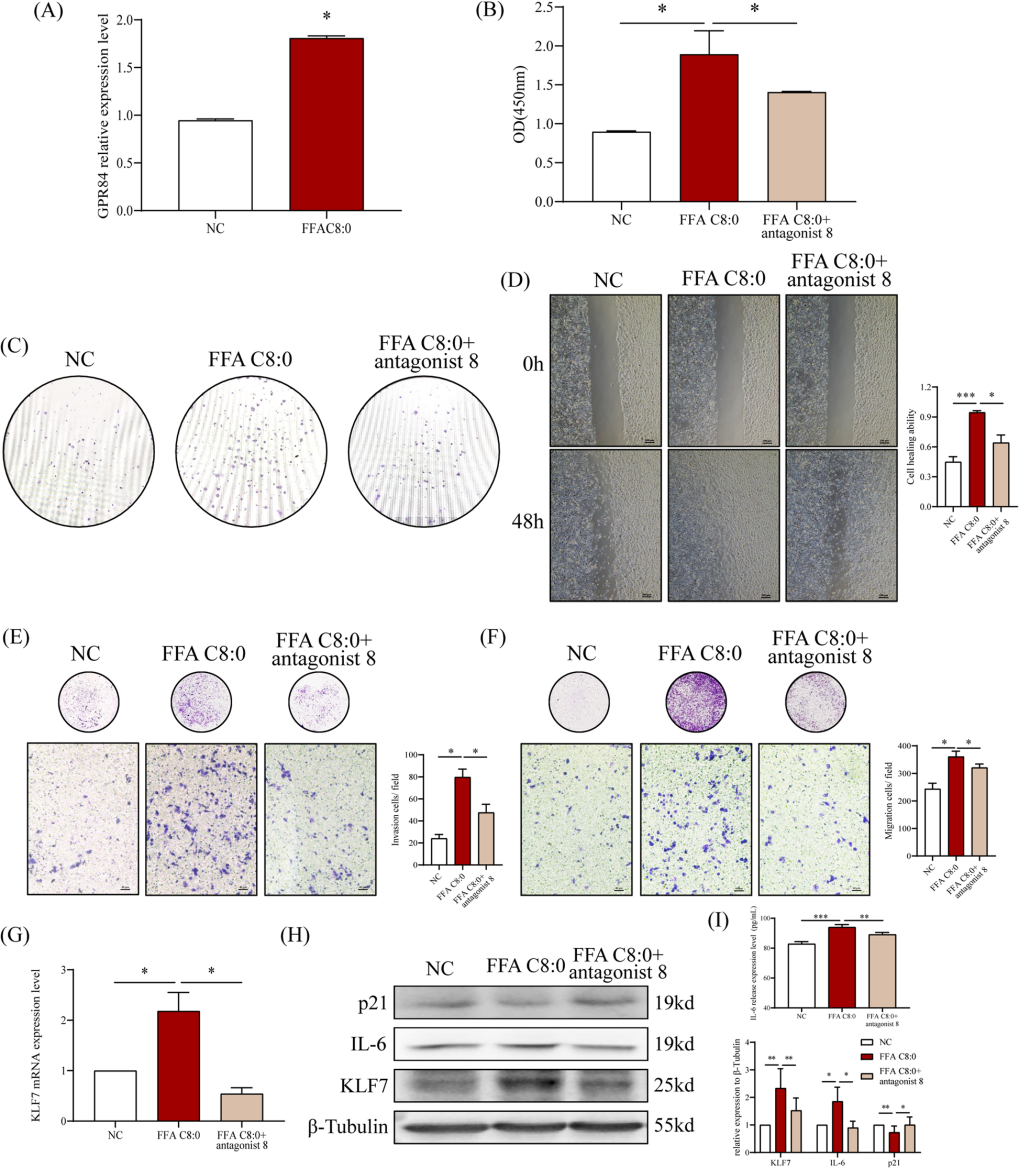
Effects of antagonize GPR84 under 200 μM FFA C8:0 stimulation on biological behavior of PC3 cells. **A **The mRNA expression level of GPR84 in PC3 cells after 200 μM FFA C8:0 stimulation was detected by qRT-PCR. **B** CCK8 assay was used to detect the proliferation ability of PC3 cells after antagonizing GPR84 under 200 μM FFA C8:0 stimulation. **C** The effect of antagonizing GPR84 under 200 μM FFA C8:0 stimulation on the colony formation ability of PC3 was detected by plate Colony assay. **D** The effect of antagonizing GPR84 under 200 μM FFA C8:0 stimulation on the healing ability of PC3 cells was detected by scratch experiment (40 ×). **E**–**F** The effect of antagonizing GPR84 under 200 μM FFA C8:0 stimulation on the invasion (**E**) and migration (**F**) ability of PC3 cells was detected by Transwell assay (200 ×). **G** The mRNA expression level of KLF7 in PC3 cells after antagonizing GPR84 under 200 μM FFA C8:0 stimulation was detected by qRT-PCR. **H** The protein expression levels of KLF7, IL-6, and p21 in PC3 cells after antagonizing GPR84 under 200 μM FFA C8:0 stimulation were detected by Western Blot. **I** The secretion level of IL-6 in PC3 cells after antagonizing GPR84 under 200 μM FFA C8:0 stimulation were detected by ELISA. *one-way ANOVA* test, *P < 0.05, ***P < 0.001 the difference was statistically significant

As a medium-chain fatty acid, FFA C8:0 plays a signal transduction role through its receptor GPR84 [15, 29]. We found high expression of GPR84 in the tumor tissues of PCa patients (Supplementary Fig. 1C), the mRNA expression level of GPR84 was significantly increased in PC3 cells after being treated with high concentration of FFA C8:0 (Fig. 6A). To explore whether FFA C8:0 could up-regulate KLF7 and promote PCa via GPR84, we stimulated PC3 cells with FFA C8:0 while adding GPR84 antagonist 8. The results showed that after blocking GPR84, the proliferation, colony formation, scratch healing, invasion, and migration abilities of PC3 cells enhanced by FFA C8:0 were significantly weakened (Fig. 6B-F). After treatment with GPR84 antagonist 8, the expression levels of KLF7 and IL-6 were significantly reduced, while p21 was increased (Fig. 6G-I). We discovered that FFA C8:0 promoting the development of PCa by up-regulating KLF7, GPR84 plays an important role in this process.

## Discussion

Epidemiological studies have shown that obesity is closely related to the occurrence of high-grade malignant PCa [30, 31]. After obesity, adipose tissue expands abnormally, lipolysis increases, and a large number of free fatty acids and adipokines are released into the circulation [32, 33]. The released FFAs not only provides energy for tumor tissues but also function as signal transduction factor by participating in a variety of regulation mechanisms of tumor occurrence and development [34, 35]. FFAs can be classified into different types, according to the carbon chain length and structure. Different types of FFAs exert different functions in the development of PCa [9, 36].

In the previous study, we found the concentrations of FFA C8:0 were increase in the serum of patients with PCa, especially PCa bone metastasis patients, but the mechanism that FFA C8:0 participates in the progress of PCa is unclear [17]. In this study, two types of PCa cells with different levels of malignancy were treated with different concentrations of FFA C8:0, results showed that the proliferation, colony formation, invasion, and migration capabilities of PCa cells were significantly enhanced, androgen-independent PCa cells PC-3 were more sensitive to FFA C8:0 stimulation than 22RV1. In addition, the mRNA and protein expression levels of MMP2 increased significantly in PC-3 cells after being stimulated by high concentration of FFA C8:0.

In most cases, the interaction of androgen receptors and their ligands (such as DHT) plays a key role in the development of PCa. In the early stage of tumor development, androgens such as DHT can combine with androgen receptor with high affinity, and activate the transcription of many downstream target genes after entry nuclear to promote the proliferation and development of androgen-dependent prostate cancer cells (such as 22RV1). In the castration-resistant prostate cancer (such as PC3), due to the mutation of androgen receptor, many other signaling pathways began to replace androgen receptor-related pathways to promote the progress of PCa [37]. As pointed out by Abdulghani et al., long-chain fatty acids can enter the castration-resistant prostate cancer cells through fatty acid-binding protein 5 to activate peroxisome proliferator-activated receptor γ as signal molecules, then regulate downstream target genes to promote the progress of PCa [38]. In this study, we found PC3 and 22RV1 cells have different sensitivity to FFA C8:0, which may be related to the above literature.

KLF7 is a zinc finger transcription factor, which plays an important role in cell proliferation, differentiation, and lipid synthesis. In recent years, KLF7 has been found to play an important role in regulating inflammatory response and tumor progression [39–42]. However, there is no research about the relationship between KLF7 and PCa. In this study, KLF7 was highly expressed in both PCa tissues and obese primary PCa-bearing mouse models. After up-regulating the expression of KLF7, it was found that the proliferation, invasion, and migration abilities of PCa cells were considerably enhanced. Furthermore, PCa cells were treated with FFA C8:0 and interfered with KLF7 expression, results showed that FFA C8:0 could promote the occurrence of prostate cancer by regulating KLF7.

After clarifying that the transcription factor KLF7 plays an important role in the development of obesity-induced PCa, searching for key downstream target genes of KLF7 may provide important experimental evidence for the treatment and prevention of PCa. It has been reported that the inflammatory factor IL-6, which is closely related to PCa hyperproliferation, aggressive phenotype, castration resistance, and bone metastasis, and there may be a KLF7 target promoter region [23, 43, 44]. Besides, p21,as a common cyclin kinase inhibitor, plays an important role in controlling the cell cycle process, and can often play an anti-tumor cell proliferation effect by inhibiting the cell cycle [45]. It was found that there were targeted binding sites for KLF7 in the promoter region of P21 [27]. Our results indicated that FFA C8:0 further regulates IL-6/p21 expression by regulating KLF7 and participates in the occurrence and development of prostate cancer. In addition, high expression of IL-6 and low expression of p21 were found in PCa tumor tissues of primary PCa tumor-bearing mice with obese.

As a medium-chain fatty acid, FFA C8:0 often exerts molecular regulation through its fatty acid receptor GPR84 [16, 46]. In a study of acute myeloid leukemia, activated GPR84 can promote disease resistance through the Wnt /β-catenin axis, which targets and modulates the expression of factor KLF7 in non-small cell lung cancer cells [42, 47]. In this study, when PCa cells were stimulated with high levels of FFA C8:0 and GPR84 antagonist 8, the proliferation, colony formation, invasion, and migration capabilities enhanced by FFA C8:0 were significantly weakened, and the enhanced mRNA and protein expression levels of KLF7/IL-6 were significantly reduced, while the expression level of p21 was reversed.

## Conclusion

In summary, this study confirmed elevated levels of FFA C8:0 in obesity can up-regulate the transcription factor KLF7 through fatty acid receptor GPR84. The up-regulated KLF7 promotes the expression of IL-6 and inhibits the expression of p21, then ultimately promotes the development of PCa. The results of this study will provide a new basis for the prevention and treatment of PCa.

## Supplementary Information

**Additional file 1: Supplementary Table 1.** Primer Sequences (to Methods- Quantitative Real‐Time PCR). **Supplementary Table 2.** Patient characteristic (to Result1). **Supplementary Table 3.** Comparison of the tumor formation rate of prostate cancer cells in normal diet and high-fat diet mice (to Result1). **Supplementary Table 4.** Glucose and lipid levels in serum of mice under High-Fat Diet (to Result1). **Supplementary Fig. 1.** Correlation between KLF7 and other factors in tumor tissues of patients with PCa (to Result1). **Supplementary Fig. 2.** The basic expression level of KLF7/GPR84 in PC3 and 22RV1 cells.

**Additional file 2.** Original full-length gel and blot images.

**Additional file 3.** ARRIVE checklist. Details of animal experiments.

## Data Availability

We have submitted part of the original data in the study to the system as supplementary materials. If you need other original data involved in the manuscript, please contact us at any time.

## Abbreviations

PCa: Prostate cancer
BPH: Benign prostatic hyperplasia
FFAs: Free fatty acids
FFA C8:0: Caprylic Acid
GPR84: G protein-coupled receptor 84
KLF7: Krüppel-like factor 7
Ki67: Nuclear-associated Antigen
MMP2: Matrix Metalloproteinase 2
