# Supplementary material for: Caprylic Acid (FFA C8:0) promotes the progression of prostate cancer by up-regulating G protein-coupled receptor 84/ Krüppel-like factor 7
Source: BMC Cancer. 2023 May 11;23:426. doi: 10.1186/s12885-023-10841-2 (PMC10173472; doi:10.1186/s12885-023-10841-2)
Supplement: Supplementary file 1 — Additional file 1: Supplementary Table 1. Primer Sequences (to Methods- Quantitative Real‐Time PCR). Supplementary Table 2. Patient characteristic (to Result1). Supplementary Table 3. Comparison of the tumor formation rate of prostate cancer cells in normal diet and high-fat diet mice (to Result1). Supplementary Table 4. Glucose and lipid levels in serum of mice under High-Fat Diet (to Result1). Supplementary Fig. 1. Correlation between KLF7 and other factors in tumor tissues of patients with PCa (to Result1). Supplementary Fig. 2. The basic expression level of KLF7/GPR84 in PC3 and 22RV1 cells. [file 12885_2023_10841_MOESM1_ESM.docx]

**Supplementary table 1 Primer Sequences**

| Primer | Sequences (5ʹ→3ʹ) | Fragment |
| --- | --- | --- |
| *Human-KLF7-F* | CTCAATGGTGGTGCTTGCTT | 233bp |
| *Human-KLF7-R* | TGGAAAACCTGCTCGCTCTA |  |
| *Human-IL-6-F* | AGACAGCCACTCACCTCTTCAG | 132bp |
| *Human-IL-6-R* | TTCTGCCAGTGCCTCTTTGCTG |  |
| *Human-p21-F* | AGGTGGACCTGGAGACTCTCAG | 194bp |
| *Human-p21-R* | TCCTCTTGGAGAAGATCAGCCG |  |
| *Human-GPR84-F* | CTCCAGAAGCATCTGCCAAAGC | 116bp |
| *Human-GPR84-R* | GGCAAAGCAGAGGAACACAGCA |  |
| *Human-MMP2-F* | AGCGAGTGGATGCCGCCTTTAA | 138bp |
| *Human-MMP2-R* | CATTCCAGGCATCTGCGATGAG |  |
| *Human- GAPDH-F* | GGTGGTCTCCTCTGACTTCAA | 211bp |
| *Human-GAPDH-R* | TCTTCCTCTTGTGCTCTTGCT |  |
| KLF7 (si-1)  KLF7-Homo-555 | GCCUUGAAUUGGAACGCUATT |  |
|  | UAGCGUUCCAAUUCAAGGCTT |  |
| KLF7 (si-2)  KLF7-Homo-611 | GGUGAGGACUUGGACUGUUTT |  |
|  | AACAGUCCAAGUCCUCACCTT |  |

**Supplementary table 2. Patient characteristic (Mean±Std. Deviation)**

|  | **BPH(n=30)** | **PCa(n=30)** |
| --- | --- | --- |
| **Age** | 72±5.32 | 75.37±5.59 |
| **Height(cm)** | 165.1±6.71 | 164.83±6.12 |
| **Weight（kg）** | 64.1±12.08 | 65.62±9.94 |
| **BMI** | 23.45±3.71 | 24.07±3.58 |
| **TC(mmol/L)** | 4.28±1 | 3.37±1.92* |
| **TG(mmol/L)** | 1.14±0.59 | 2.58±1.92*** |
| **LDL(mmol/L)** | 2.64±0.86 | 2.72±1.24 |
| **HDL(mmol/L** | 1.12±0.3 | 1.16±0.33 |
| **GLU(mmol/L)** | 5.72±1.75 | 6.16±1.92 |
| **PSA(mmol/L)** | 5.2±6.03 | 50.19±38.8*** |

*t* test, ^*^*P* <0.05, ^***^*P*<0.01 the difference was statistically significant

**Supplementary tabal 3. Comparison of the tumor formation rate of**

**prostate cancer cells in normal diet and high-fat diet mice**

| Group | Tumor formation | Non-tumorous | Total | Tumor formation rate |
| --- | --- | --- | --- | --- |
| NCD | 3 | 2 | 5 | 60% |
| HFD | 9 | 1 | 10 | 90% |
| Total | 12 | 3 | 15 | 80% |

*Chi-square* test, *χ*^2^ =5.4, *P*=0.01, the difference was statistically significant

**Supplementary table 4.**

**Glucose and lipid levels in serum of mice under High-Fat Diet (Mean ±SD)**

|  | FFA(mmol/L) | TG(mmol/L) | TC(mmol/L) | HDL(mmol/L) | LDL(mmol/L) | | GLU(mmol/L) |
| --- | --- | --- | --- | --- | --- | --- | --- |
| NCD (n=5) | 0.56±0.34 | 0.50±0.15 | 4.27±1.43 | 0.47±0.16 | | 0.22±0.18 | 9.92±3.63 |
| HFD  (n=10) | 0.93±4.48^**^ | 0.70±0.25^**^ | 5.77±0.83^***^ | 0.44±0.15 | | 0.18±0.15 | 11.1±5.17 |

*Non-parametric rank sum* test, ^**^*P*<0.01, ^***^*P*<0.01 the difference was statistically significant

**Supplementary Figure 1：**


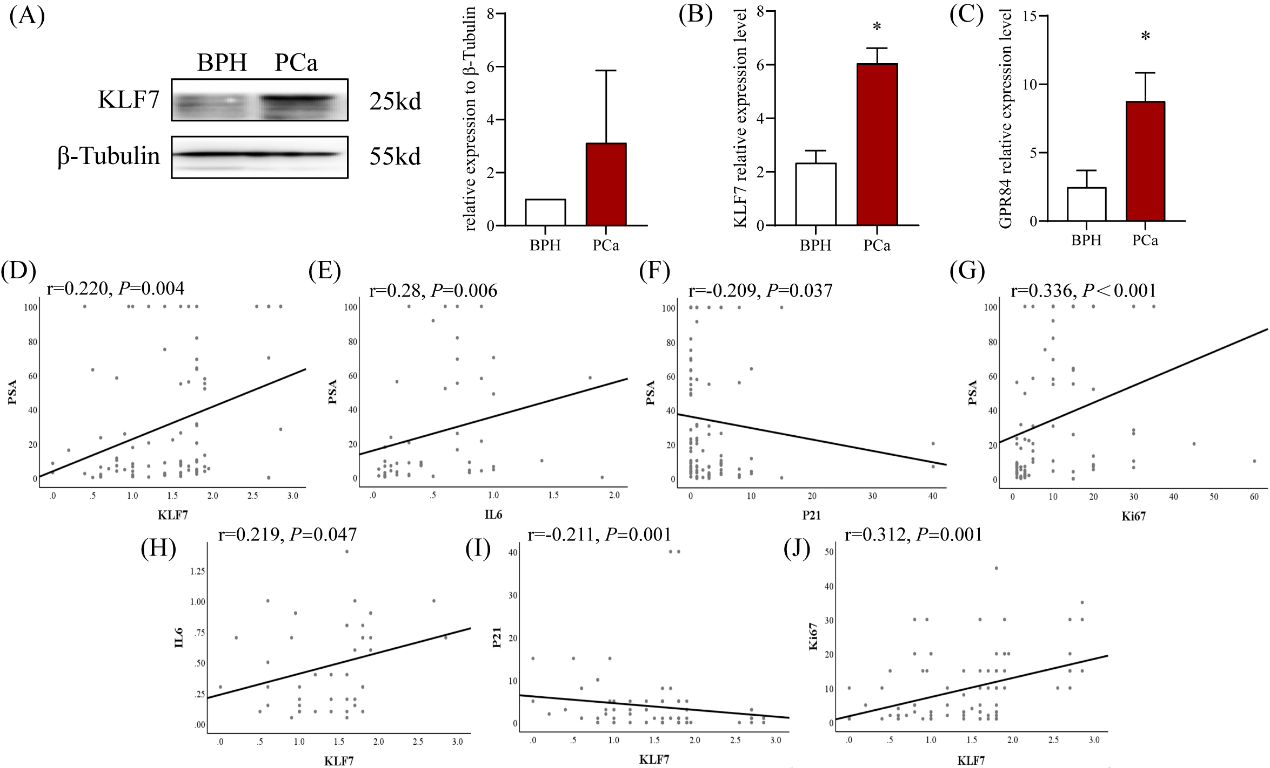


**Supplementary Figure 1. Correlation between KLF7 and other factors in tumor tissues of patients with PCa.**

(A) Western Blot was used to detect the protein expression level of KLF7 in BPH tissues and tumor tissues with PCa. (B-C) qRT-PCR was used to detect the mRNA expression level of KLF7(B) and GPR84(C) in BPH tissues and tumor tissues with PCa. (D-G) Pearson method was used to analyze the correlation between serum PSA in PCa patients and the expression levels of KLF7(D), IL-6(E), p21(F) and Ki67(G) in tumor tissues. (H-J) Pearson method was used to analyze the correlation between KLF7 and IL-6(H), p21(I), Ki67(J) expression levels in tumor tissues of PCa patients.

*t* test, **P*<0.05 the difference was statistically significant.

**Supplementary Figure 2：**


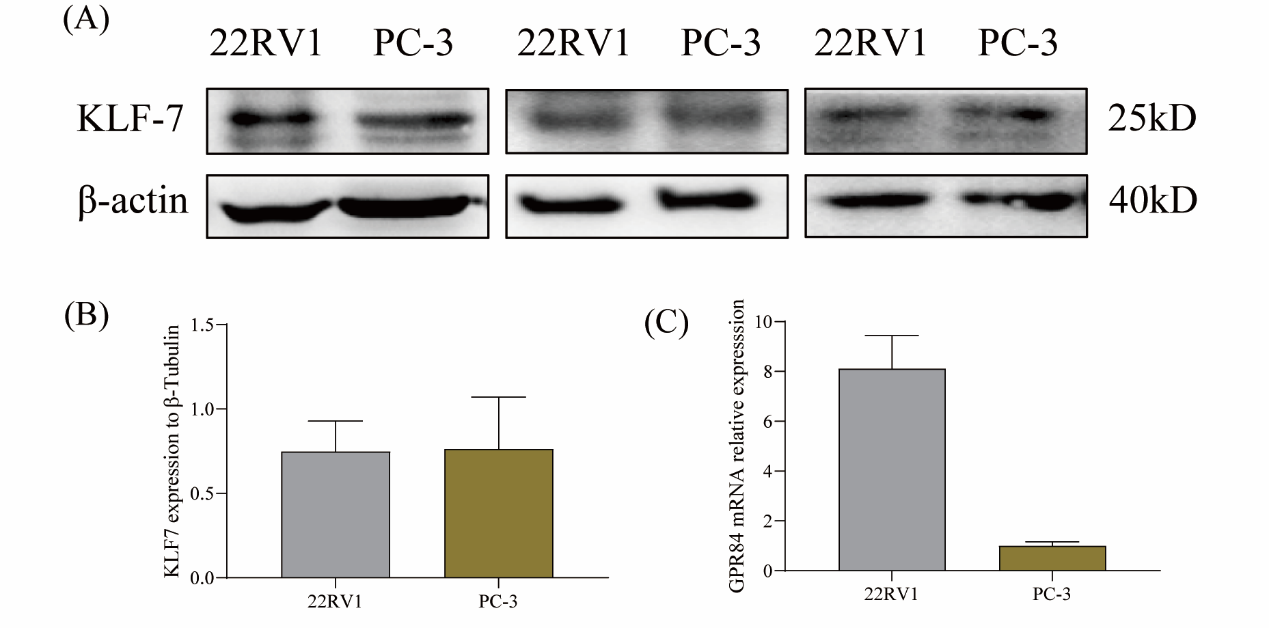


**Supplementary Figure 2.** **The basic expression level of KLF7/GPR84 in PC3 and 22RV1 cells.**

(A-B) Western Blot was used to detect the protein expression level of KLF7 in 22RV1 and PC3 cells. (C) qRT-PCR was used to detect the mRNA expression level of GPR84 in 22RV1 and PC3 cells.
