# Supplementary material for: Caprylic Acid (FFA C8:0) promotes the progression of prostate cancer by up-regulating G protein-coupled receptor 84/ Krüppel-like factor 7
Source: BMC Cancer. 2023 May 11;23:426. doi: 10.1186/s12885-023-10841-2 (PMC10173472; doi:10.1186/s12885-023-10841-2)
Supplement: Supplementary file 2 — Additional file 2. Original full-length gel and blot images. [file 12885_2023_10841_MOESM2_ESM.pptx]

## Slide 1
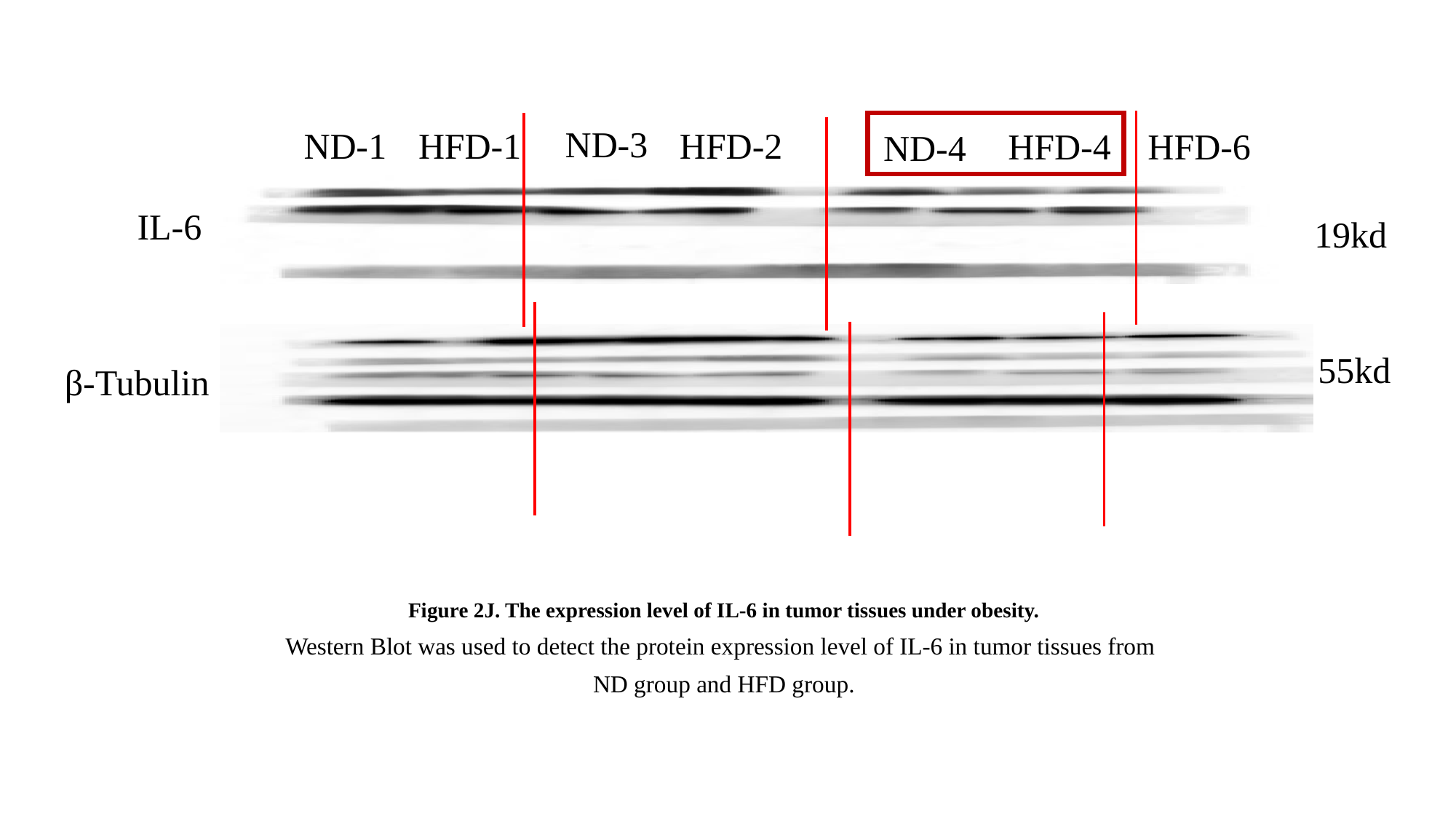

ND-3
ND-1
HFD-1
HFD-2
HFD-4
HFD-6
ND-4
IL-6
19kd
55kd
β-Tubulin
Figure 2J. The expression level of IL-6 in tumor tissues under obesity.
Western Blot was used to detect the protein expression level of IL-6 in tumor tissues from
ND group and HFD group.

## Slide 2
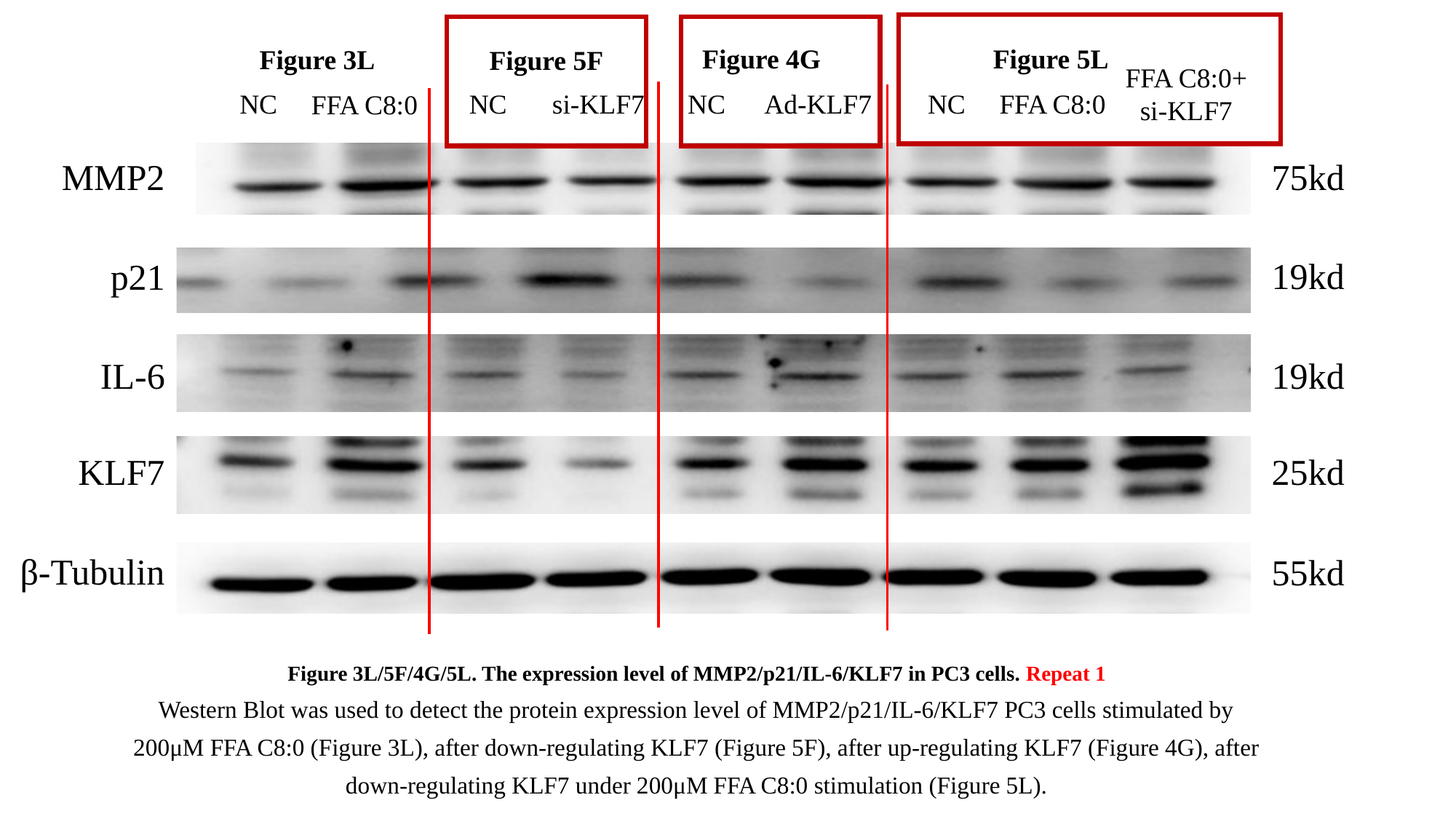

Figure 4G
Figure 5L
Figure 3L
Figure 5F
FFA C8:0+
si-KLF7
NC
NC
NC
si-KLF7
NC
Ad-KLF7
FFA C8:0
FFA C8:0
MMP2
75kd
19kd
p21
19kd
IL-6
KLF7
25kd
β-Tubulin
55kd
Figure 3L/5F/4G/5L. The expression level of MMP2/p21/IL-6/KLF7 in PC3 cells. Repeat 1
Western Blot was used to detect the protein expression level of MMP2/p21/IL-6/KLF7 PC3 cells stimulated by 200μM FFA C8:0 (Figure 3L), after down-regulating KLF7 (Figure 5F), after up-regulating KLF7 (Figure 4G), after down-regulating KLF7 under 200μM FFA C8:0 stimulation (Figure 5L).

## Slide 3
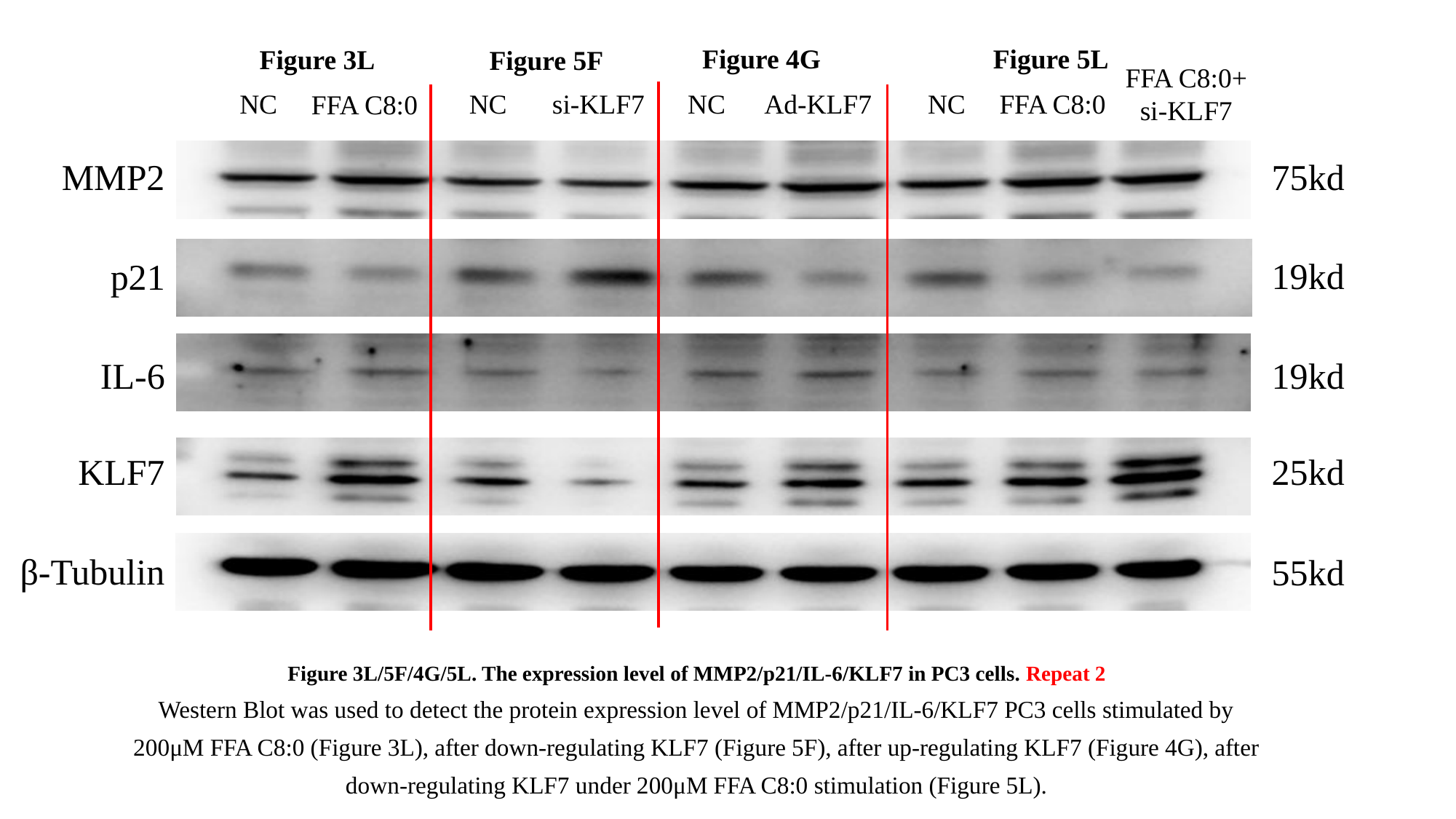

Figure 4G
Figure 5L
Figure 3L
Figure 5F
FFA C8:0+
si-KLF7
NC
NC
NC
si-KLF7
NC
Ad-KLF7
FFA C8:0
FFA C8:0
MMP2
75kd
19kd
p21
19kd
IL-6
KLF7
25kd
β-Tubulin
55kd
Figure 3L/5F/4G/5L. The expression level of MMP2/p21/IL-6/KLF7 in PC3 cells. Repeat 2
Western Blot was used to detect the protein expression level of MMP2/p21/IL-6/KLF7 PC3 cells stimulated by 200μM FFA C8:0 (Figure 3L), after down-regulating KLF7 (Figure 5F), after up-regulating KLF7 (Figure 4G), after down-regulating KLF7 under 200μM FFA C8:0 stimulation (Figure 5L).

## Slide 4
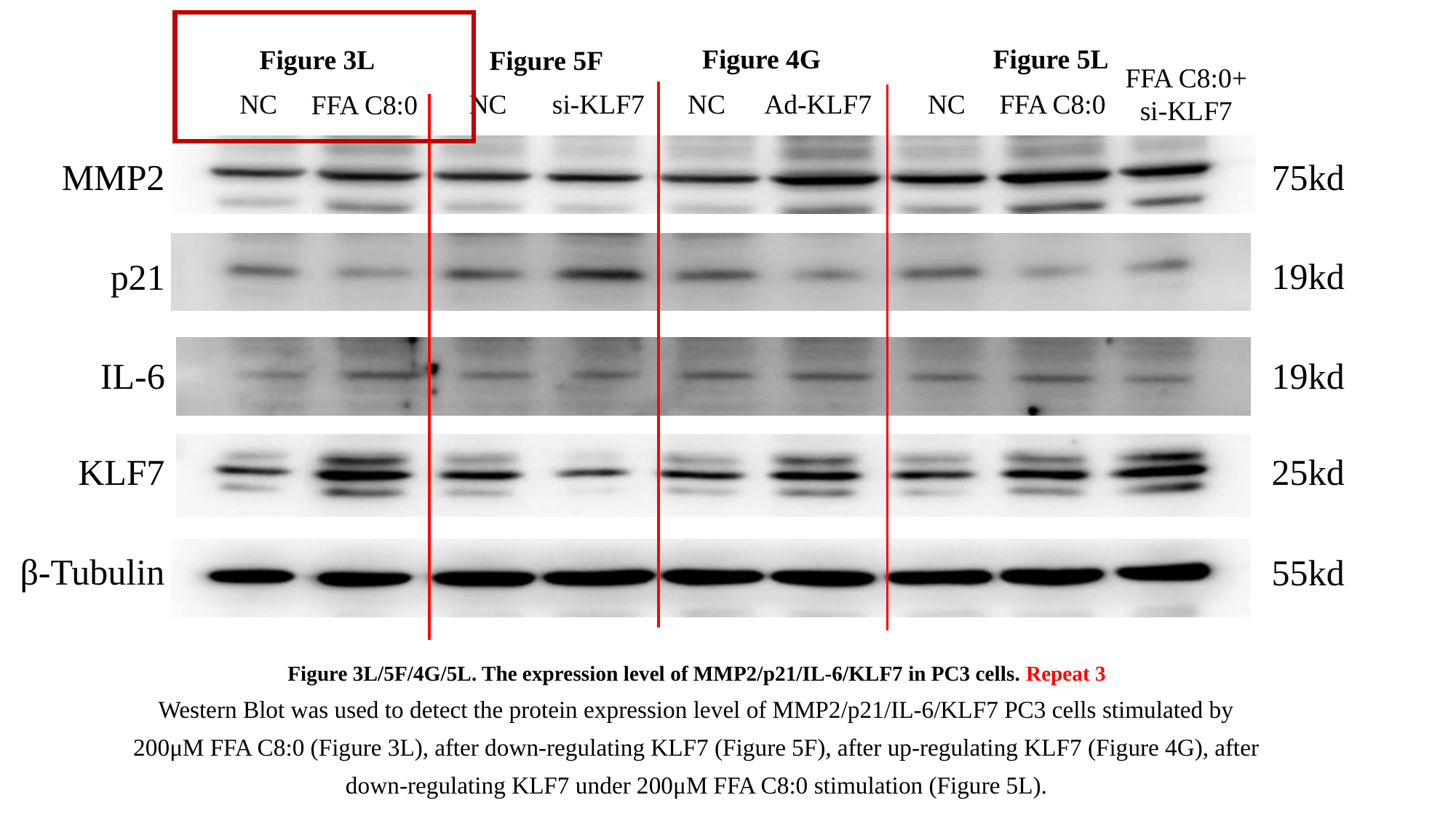

Figure 4G
Figure 5L
Figure 3L
Figure 5F
FFA C8:0+
si-KLF7
NC
NC
NC
si-KLF7
NC
Ad-KLF7
FFA C8:0
FFA C8:0
MMP2
75kd
19kd
p21
19kd
IL-6
KLF7
25kd
β-Tubulin
55kd
Figure 3L/5F/4G/5L. The expression level of MMP2/p21/IL-6/KLF7 in PC3 cells. Repeat 3
Western Blot was used to detect the protein expression level of MMP2/p21/IL-6/KLF7 PC3 cells stimulated by 200μM FFA C8:0 (Figure 3L), after down-regulating KLF7 (Figure 5F), after up-regulating KLF7 (Figure 4G), after down-regulating KLF7 under 200μM FFA C8:0 stimulation (Figure 5L).

## Slide 5
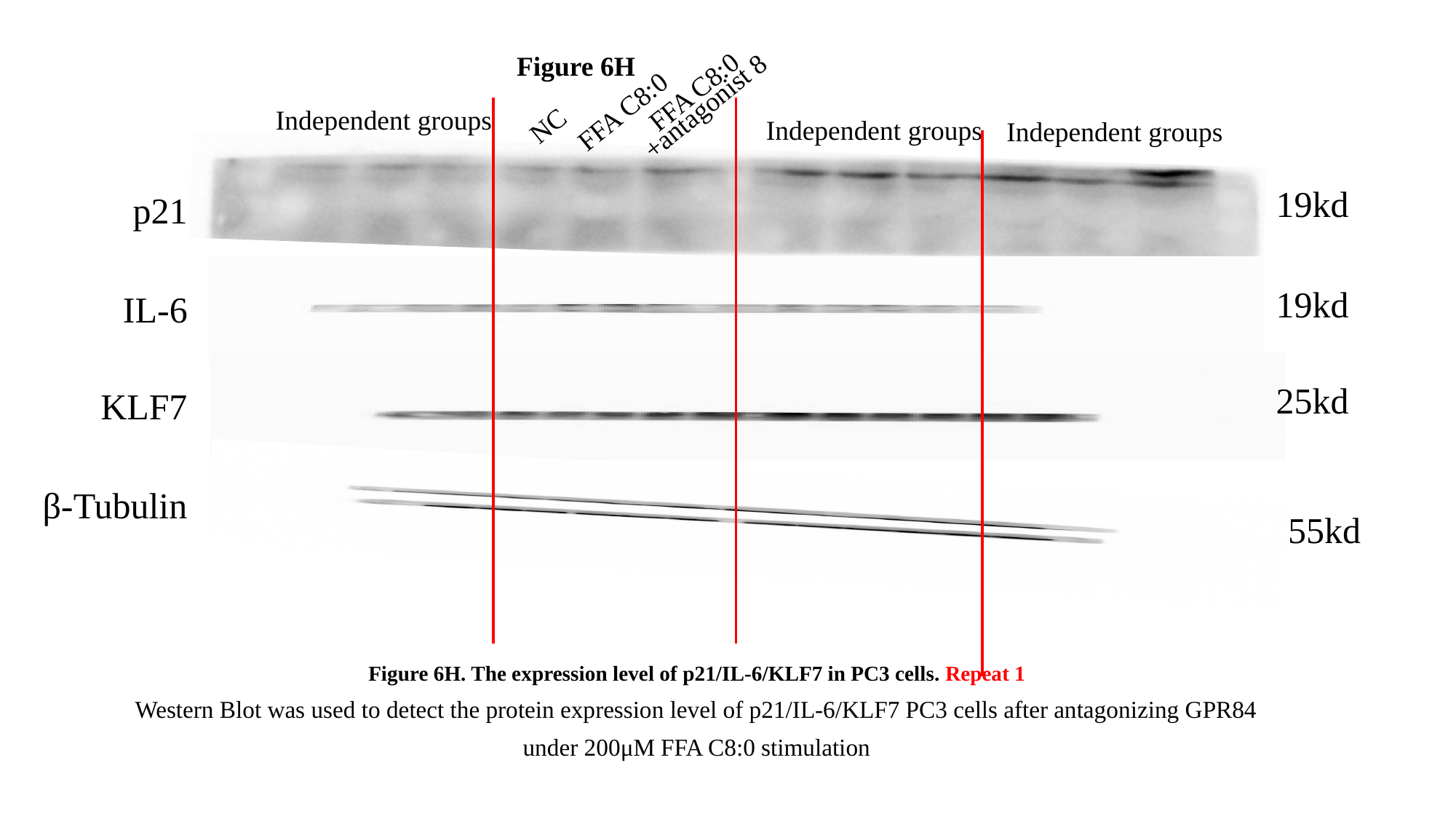

Figure 6H
FFA C8:0
+antagonist 8
FFA C8:0
Independent groups
NC
Independent groups
Independent groups
19kd
p21
19kd
IL-6
25kd
KLF7
β-Tubulin
55kd
Figure 6H. The expression level of p21/IL-6/KLF7 in PC3 cells. Repeat 1
Western Blot was used to detect the protein expression level of p21/IL-6/KLF7 PC3 cells after antagonizing GPR84 under 200μM FFA C8:0 stimulation

## Slide 6
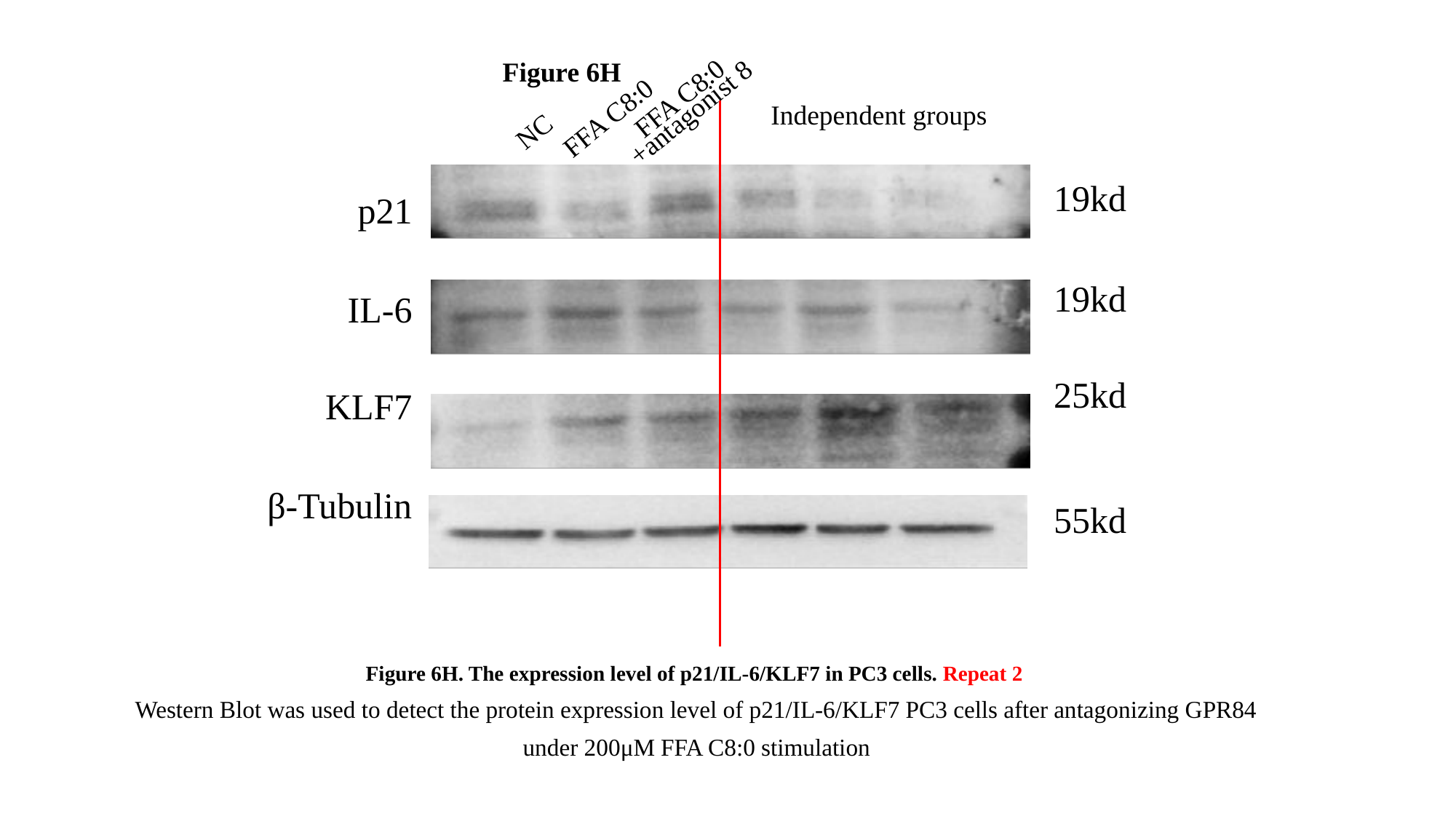

Figure 6H
FFA C8:0
+antagonist 8
FFA C8:0
NC
19kd
p21
19kd
IL-6
25kd
KLF7
β-Tubulin
55kd
Independent groups
Figure 6H. The expression level of p21/IL-6/KLF7 in PC3 cells. Repeat 2
Western Blot was used to detect the protein expression level of p21/IL-6/KLF7 PC3 cells after antagonizing GPR84 under 200μM FFA C8:0 stimulation

## Slide 7
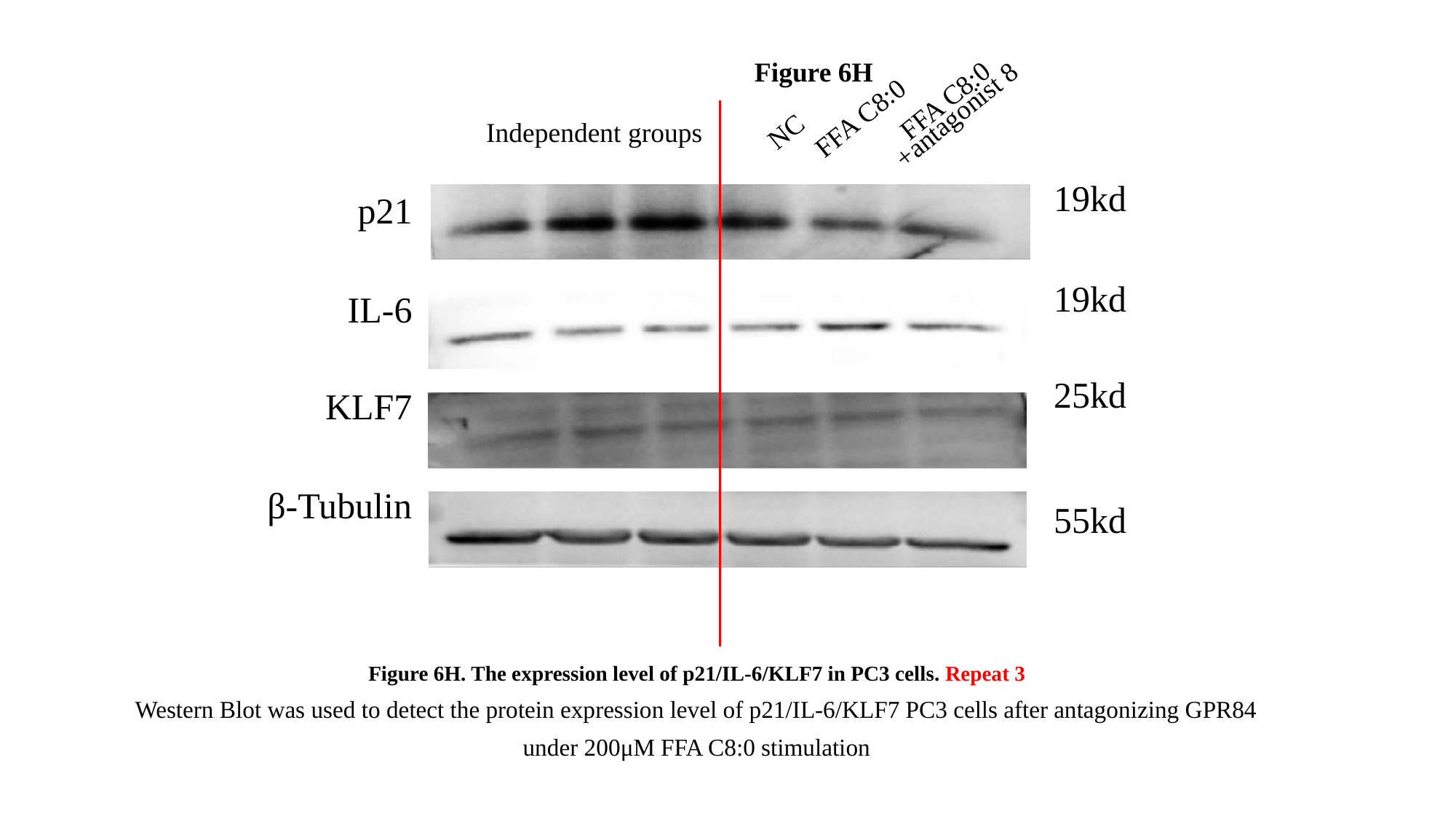

Figure 6H
FFA C8:0
+antagonist 8
FFA C8:0
NC
Independent groups
19kd
p21
19kd
IL-6
25kd
KLF7
β-Tubulin
55kd
Figure 6H. The expression level of p21/IL-6/KLF7 in PC3 cells. Repeat 3
Western Blot was used to detect the protein expression level of p21/IL-6/KLF7 PC3 cells after antagonizing GPR84 under 200μM FFA C8:0 stimulation

## Slide 8
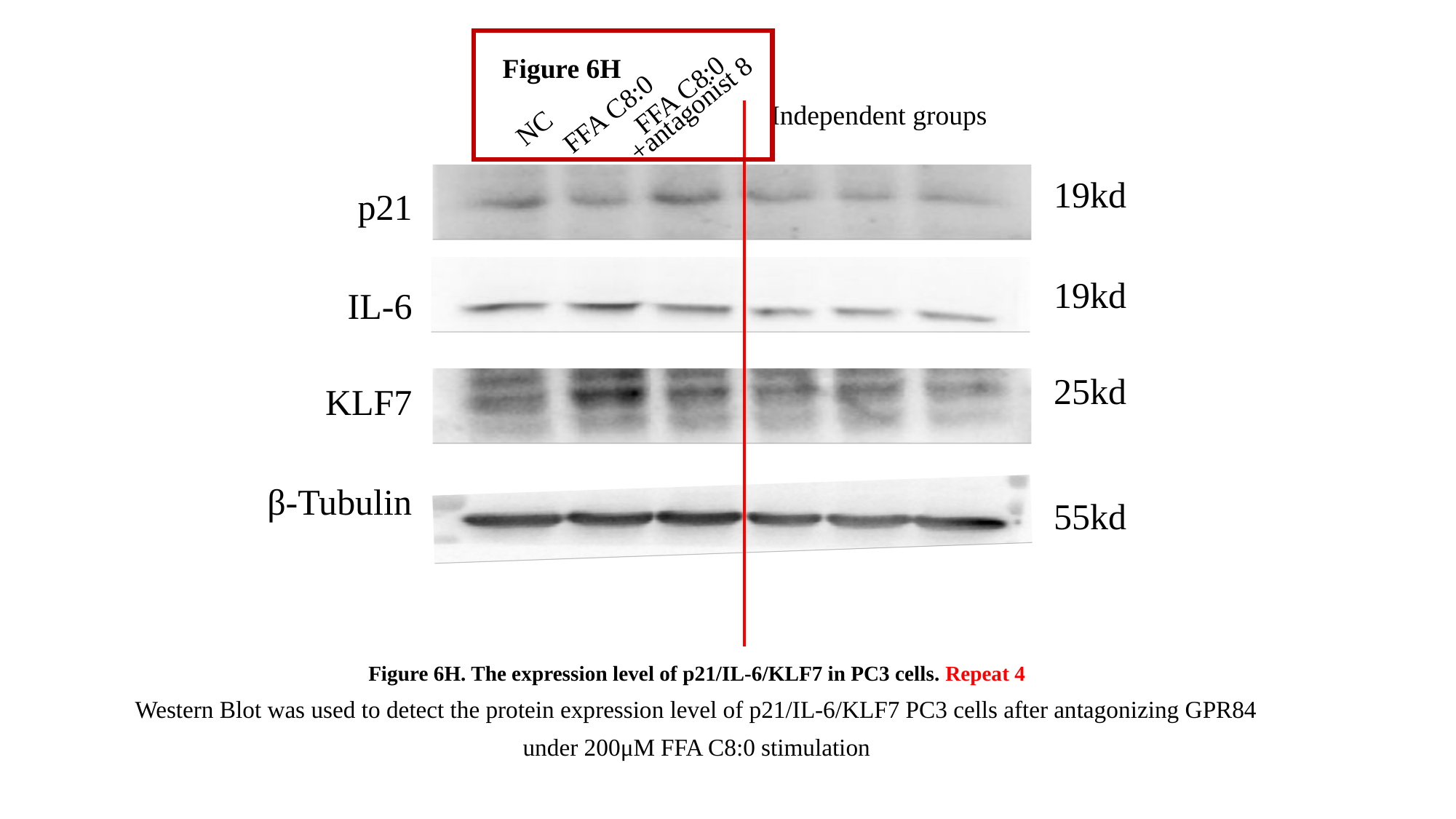

Figure 6H
FFA C8:0
+antagonist 8
FFA C8:0
Independent groups
NC
19kd
p21
19kd
IL-6
25kd
KLF7
β-Tubulin
55kd
Figure 6H. The expression level of p21/IL-6/KLF7 in PC3 cells. Repeat 4
Western Blot was used to detect the protein expression level of p21/IL-6/KLF7 PC3 cells after antagonizing GPR84 under 200μM FFA C8:0 stimulation

## Slide 9
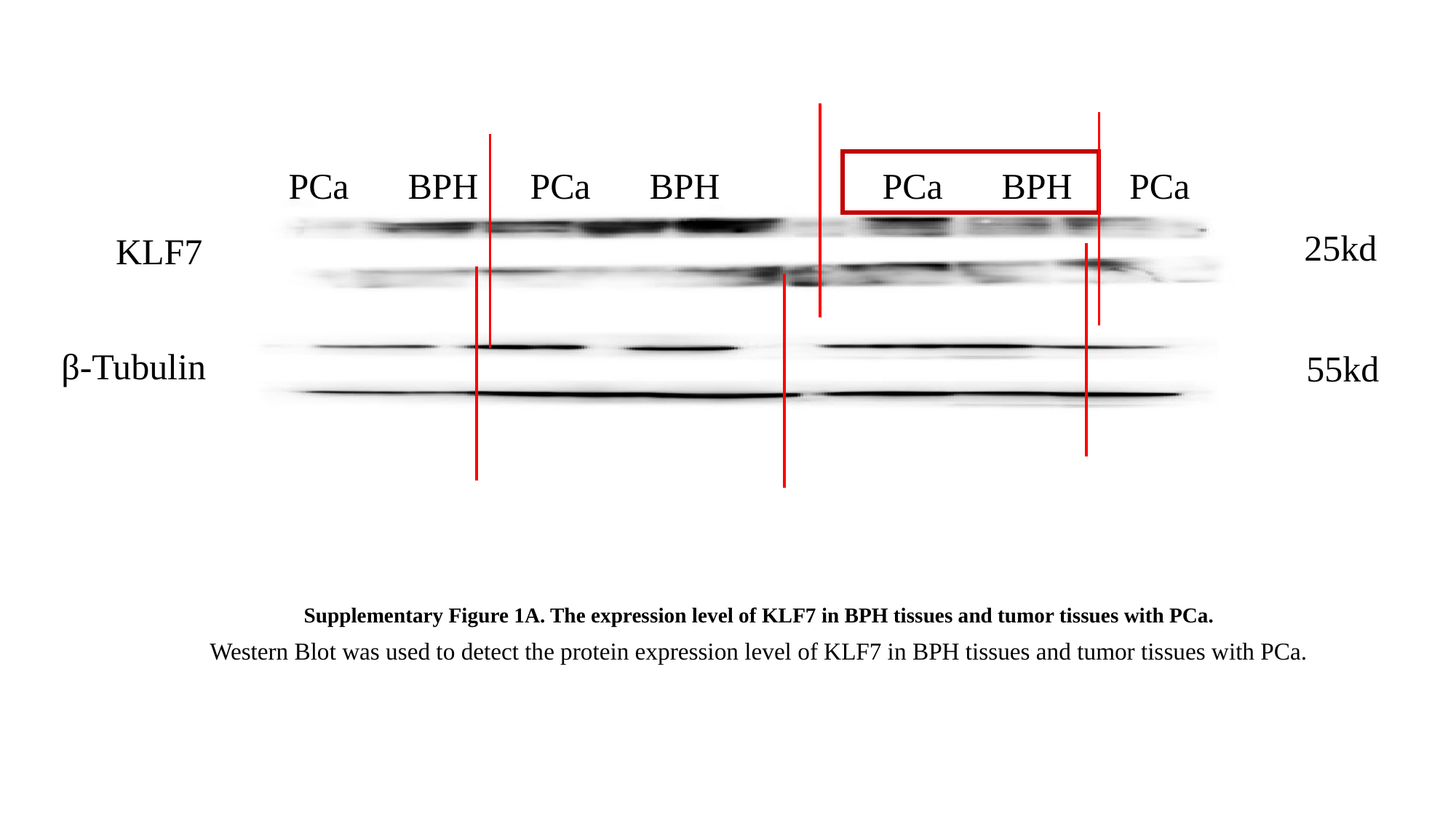

PCa
BPH
PCa
BPH
PCa
BPH
PCa
25kd
KLF7
β-Tubulin
55kd
Supplementary Figure 1A. The expression level of KLF7 in BPH tissues and tumor tissues with PCa.
Western Blot was used to detect the protein expression level of KLF7 in BPH tissues and tumor tissues with PCa.
