# Supplementary material for: Caprylic Acid (FFA C8:0) promotes the progression of prostate cancer by up-regulating G protein-coupled receptor 84/ Krüppel-like factor 7
Source: BMC Cancer. 2023 May 11;23:426. doi: 10.1186/s12885-023-10841-2 (PMC10173472; doi:10.1186/s12885-023-10841-2)
Supplement: Supplementary file 3 — Additional file 3. ARRIVE checklist. Details of animal experiments. [file 12885_2023_10841_MOESM3_ESM.docx]

**ARRIVE checklist**

**1. Study Design (Page4, Methods, paragraph 2)**

To investigate whether the high expression of KLF7 in PCa tumors is related to obesity-induced PCa, male BALB/ C nude mice were fed with high-fat diet (HFD, 60% fat Kcal%) to construct a mice obesity model.

Test group: Fed High-Fat Diet (HFD, 60% fat Kcal%, Medicine, Jiangsu, China)

Control group: Normal Diet (ND, 10% fat Kcal%, Medicine, Jiangsu, China

After the two groups of mouse showed significant differences in body weight and Lee`s, 5×10^5^ PC3-Luc cells were injected into the prostate of each mice[1].

**2. Sample size (Page4, Methods, paragraph 2)**

In order to reduce the number of animal sacrifices and ensure the success rate of operation in experimental mice, we adopted a small sample size design.

Test group: n=10

Control group: n=5

**3. Inclusion and exclusion criteria (Page4, Methods, paragraph 2)**

Mice were included in the study if they survived surgery after in-situ injection of PC3 cells into the prostate. Mice that died or were unable to eat or move normally after surgery were excluded prematurely.

**4. Randomisation (Page4, Methods, paragraph 2)**

All male mice were randomly assigned to Test group and Control group before group feeding, and then fed in groups of 5 in a cage. Among them, 10 mouse in Test group were randomly assigned to 2 cages and fed with high fat.

To ensure the normal survival of mouse, mouse in both groups ate freely.

**5. Blinding (Page4, Methods, paragraph 3)**

Four researchers participated in the animal study. The first researcher was responsible for group feeding and numbering of the mice, the second researcher was responsible for in-situ prostate injection surgery (the group of mice was unknown), the third researcher was responsible for vivo imaging and surgical dissection of tumor tissue from the mice (the group of mice was unknown), the fourth researcher was responsible for analyzing the tumor volume, weight and expression levels of various factors in the tissue according to the number (the group of mice was unknown), and sent the data to the first researcher for analysis.

**6. Outcome measures (Page8, Result 1, paragraph 3)**

In this experiment, the body weight and body length of mice were dynamically detected throughout the whole process. Finally, the tumor formation of PCa cells in mouse prostate was observed, tumor volume and weight were collected, FFA/TG/TC/HDL/LDL/GLU in serum were detected, and protein expression levels of various factors in tumor tissues were detected.

**7. Statistical methods (Page8, Methods, paragraph 16)**

SPSS (v. 17.0) computer software was used for all statistical analysis. Mean and standard deviation were determined as the main parameters, and the average of data between the experimental and control groups were compared using *Non-parametric rank sum* test. Values of *P* <0.05 as a standard of significant difference.

**8. Experimental animals (Page4, Methods, paragraph 2)**

Male BALB/ C nude mice was used in this study and all mouse were 4 weeks old (Vital River, Beijing, China).

**9. Experimental procedures (Page4, Methods, paragraph 2)**

Fifteen 4-week-old male mouse were raised in the specific pathogen free animal room. All food and drinking water are strictly sterilized and free to ingest. After a week of adaptive feeding, the mice were fed High-Fat Diet and Normal Diet. After the two groups of mice showed significant differences in body weight and Lee`s, 5×10^5^ PC3-Luc cells were injected into the prostate of each mice. HFD feeding continued for 12 weeks after surgery, they were given inhalation anesthetized using isoflurane, inject D-luc substrate enzyme into the abdominal cavity and observe the PCa tumor formation within 30 minutes using a small animal in vivo imaging instrument, cervical dislocation method made mice die and surgically felt the intra-abdominal tumor tissue.

**10. Results (Page8, Result 1, paragraph 3; Page12, Figure legends-** **Figure 2, paragraph 2)**

The body length and tumor tissue volume of mice were measured with vernier calipers.

The weight and tumor tissue weight of mice were weighed by electronic scale.

In vivo small animal imager was used to observe the in-situ tumor formation of prostatic cells in mice.

Serum FFA/TG/TC/HDL/LDL/GLU in mice were detected by the kit.

Immunohistochemistry was used to detect the protein expression levels of various factors in tumor tissues.

Western Blot was used to detect the protein expression levels of each factor in tumor tissues.

References

1. Dai J, Hensel J, Wang N, Kruithof-de Julio M, Shiozawa Y: **Mouse models for studying prostate cancer bone metastasis**. *Bonekey Rep* 2016, **5**:777.
